# Reactivity of the triple task on writing processes and product in adults with dyslexia

**DOI:** 10.3389/fpsyg.2023.1112274

**Published:** 2023-10-09

**Authors:** Marina Olujić Tomazin, Jelena Kuvač Kraljević, Rui A. Alves

**Affiliations:** ^1^Faculty of Education and Rehabilitation Sciences, Department of Hearing Impairments, University of Zagreb, Zagreb, Croatia; ^2^Faculty of Education and Rehabilitation Sciences, Department of Speech and Language Pathology, University of Zagreb, Zagreb, Croatia; ^3^Faculty of Psychology and Educational Sciences, Psychology Department, University of Porto, Porto, Portugal

**Keywords:** text writing, the triple task, reactivity, writing processes, written product, adults, dyslexia

## Abstract

**Introduction:**

The triple task (TT) is a method for assessing the dynamics of writing processes. It involves three tasks in one: writing a text, responding to a sound, and reporting the process. Previous research has mostly shown that the TT does not affect the writing process or the product. However, individuals with dyslexia often show difficulties in tasks that require organization, automation, integration of multiple processes, inhibition, and shifting/cognitive flexibility. The aim of this study was therefore to investigate whether TT affects the writing process and written product differently in adults with dyslexia compared to a control group of adults with typical reading skills.

**Methods:**

Two groups of adult native Croatian speakers were included in this study: 20 adults with developmental dyslexia and 20 adults with typical reading skills; evenly distributed by: age (18–38 years), gender (13 males, 7 females per group), educational level, and nonverbal cognitive abilities. All participants wrote one text with a TT and another without. The writing of the text was tracked with a keystroke logging program – Inputlog. The two texts were compared at process and product level.

**Results and discussion:**

The results showed that measures of writing processes and text quality in the groups of adults with dyslexia and adults with typical reading skills were unlikely to be differentially affected by TT. However, in the condition without TT, the total number of characters per minute was higher, more keys were typed per minute and more words were deleted. As expected, adults with dyslexia produced shorter texts of lower quality and with more errors; they also produced fewer characters per minute, used fewer keystrokes and typed fewer.

**Conclusion:**

The study suggests that TT is unlikely to have a different impact on the writing process or written product in adults with dyslexia compared to adults with typical reading skills.

## 1. Introduction

The Triple Task (TT) was developed to assess the timing and cognitive load of various writing processes, i.e., to assess their dynamics. It was originally developed by Kellogg in 1987. According to the assumptions of Hayes and Flower’s (1980) Cognitive Process Theory of Writing, the act of writing text involves certain thinking or (meta)cognitive processes, such as planning, translating, and revising, which the writer performs in a non-linear fashion during writing process. TT is therefore used to measure the dynamics of such processes. In TT, the participant must perform three tasks more or less concurrently. In the first task, the focus is on text composing. In the second task, the participant must respond as quickly as possible to simple audio stimuli that occur in a variable interval schedule. The recorded reaction time, from which the baseline reaction time is subtracted, serves as an index of cognitive effort with respect to the first task. More precisely, this task assumes that the focus on text composing (first task) and the requirement to respond quickly to audio stimuli (second task) compete for limited attentional or working memory resources. Thus, a faster reaction time implies less cognitive effort for the ongoing cognitive process (Olive et al., 2002). In the third task, right after the second task, the participant reports on the (meta)cognitive process going on at the time of the response. The TT must be trained prior to recording because the procedure itself has to be learned.

In reviewing the literature on cognitive processes in writing, it is noticeable that not many studies use TT. Consequently, there are not many studies that have used TT in the last decade (e.g., Fidalgo et al., 2014; Limpo, 2018; Limpo and Alves, 2018), yet they have made important contribution to understanding the cognitive background of writing. There are some reasonable explanations for this “unpopularity” of TT, such as: it is a technique that is quite complex and sensitive for application; it requires some additional time for training; it is not suitable for group testing; it requires the use of custom made computer settings or software; there is no standardized response box or similar input device for use with TT, so researchers use different response techniques, for example, by voice or by clicking a mouse, response box, or keyboard key with the non-dominant hand. There is also a growing popularity of keystroke logging tools (KSL) that measure writing processes while avoiding almost all the requirements of TT. Finally, it is also important to note that the community of researchers interested in the (cognitive) processes of writing is quite small but is likely to grow thanks to KSL tools. Despite its demanding application, TT arguably provides valuable insights into the dynamics of cognitive processes in writing and thus deserves a place in studies on cognitive processes in writing. Investigating the cognitive processes involved in composing texts using TT in a population with, for example, language disorders can make an important contribution to understanding the disorder, but it can also test the potential of TT for clinical purposes. Prior to this, TT must be evaluated and validated, especially if it is being used for the first time in a population with writing difficulties.

Writing is considered one of the most complex cognitive activities, being cognitively and emotionally demanding on its own right (Kellogg, 1987). Therefore, additional tasks concurrent with writing could be reactive and disrupt the underlying cognitive processes of writing, reducing the reliability and validity of such a multiple-task method. Reactivity refers to the risk that a particular action or procedure might alter the cognitive process underlying the task (Piolat et al., 2001) or that, in a particular case, the triple task performance may disrupt or misrepresent the cognitive processes underlying the writing process and written product (Olive et al., 2002). Previous research predominantly has shown that there is no interference between the triple task (or other similar tasks such as the dual-task and the think-aloud protocols) and text quality in adults (Kellogg, 1987; Penningroth and Rosenberg, 1995; Ransdell, 1995; Piolat et al., 1996). Piolat et al. (1996) found no significant effects of coupling writing and simple reaction time task (dual-task) neither on productivity (number of words), syntactic complexity (number of words per sentence), nor on some processes: fluency, and number of revisions observed in text composing. Additionally, they did not find significant differences in the cognitive effort either on the TT or on the dual-task. Penningroth and Rosenberg (1995) compared stories written with the TT (including think-aloud protocols) and without the TT and found no differences in coherence or text quality. Finally, Piolat et al. (2001) briefly reviewed literature on the measurement reactivity and retrospective report validity in the triple task method and concluded that the TT is a stable and powerful way to measure the dynamics of written composition, it provides valid insights into writing processes and does not compromise processes or text quality. However, Janssen et al. (1996) found that think-aloud protocols alone may affect complex writing tasks more than simple ones by increasing pause length within and between sentences, and between paragraphs. Similarly, Wengelin et al. (2014) combined the keystroke logging tool (ScriptLog) and TT to analyse the reactivity of TT to writing process and product variables. The study included a sample of 40 university students who wrote expository texts under two conditions - with and without TT. The data showed no effect on product characteristics such as text length (number of words and number of characters), mean word length (number of letters per word), lexical diversity or density, or keystroke logging data such as productivity (words/min), pause frequency, pause durations and proportion of revisions. While in the condition without TT, more keystrokes and more revisions were made. More revisions, to some extent, imply also more interruptions of episodes of language production. It would be valuable to observe also whether the TT condition alters the frequency or duration of bursts [R-or P-bursts, i.e., an episode of language production terminated by a revision (R) or pause (P)]. More specifically, the “beep” sound that signals the requirement to switch the attention on a task other than writing occasionally and unnaturally interrupts the writing process, which could increase the frequency and decrease the average duration of bursts.

Special care should be taken when applying such a complex method for assessing writing to a population that struggle with reading and writing, such as people with dyslexia do. While dyslexia has traditionally been defined as a discrepancy between an average or higher general cognitive ability and low reading performance, more recent understandings of dyslexia suggest that it encompasses developmental language difficulties that go well beyond reading difficulties, including difficulties with phonological processing, word use, spelling, and writing that persist into adulthood (e.g., Lefly and Pennington, 1991; Shaywitz et al., 1992; Bruck, 1993; Ramus, 2003; Ramus et al., 2003; Smith-Spark et al., 2003; Miles and Miles, 2004; Connelly et al., 2006; Moojen et al., 2020). Moreover, Berninger et al. (2006a) demonstrated that students often manage to compensate for their reading difficulties following dyslexia language treatment, so much so that they no longer appear to have a serious problem with reading, while writing difficulties persist. In the International Classification of Diseases 11th – ICD 11 (WHO, 2019/2021), dyslexia is classified as Developmental learning disorder with impairment in reading (6A03.0), with significant and persistent difficulties manifesting in learning academic skills related to reading.

Studies dealing with language disabilities and writing are still very product-oriented and more interested in the developmental perspective, with children being the participants rather than high education students or adults. To date, there have been very few process-oriented studies of the writing of individuals with dyslexia. Studies of writing by adults with dyslexia have shown that: students with dyslexia made more morphosyntactic spelling errors, memory-related errors, punctuation errors, and capitalisation errors than the control group and that the quality of their writing was poorer based on text structure (Tops et al., 2013); adults with dyslexia wrote more slowly, produced shorter essays, used more monosyllabic and fewer polysyllabic words (possibly indicating limited word choice), and produced text with more spelling errors (Sterling et al., 1998); students with dyslexia generally produced poorer texts, as measured by a holistic essay quality assessment, and produced more spelling errors; in addition, students with dyslexia showed poorer performance on ‘lower order skills’ such as spelling and handwriting fluency (Connelly et al., 2006). Torrance et al. (2016) showed in their process- and product-oriented study that high school students with dyslexia produced four times more spelling errors than the control group and had poorer text quality at the lexical, structural, and thematic levels; they also had longer inter-key-press latencies at all text locations at the pre-word, within-word, and word levels. Wengelin (2007) showed in her KSL study that adults with dyslexia paused more frequently during writing, especially at the within-word level, and made a higher proportion of spelling-related revisions; they also demonstrated a narrower vocabulary, as measured by the lexical density and lexical diversity of the texts.

Therefore, for adults with dyslexia, writing itself is more challenging and the TT might be more reactive, i.e., more disruptive to writing processes than in adults with typically developed reading and writing skills; and not just because of reading and writing difficulties, but also abilities and/or skills underlying the mentioned language skills. Coordinating multiple processes when composing texts, i.e., focusing and shifting attention between (meta)cognitive processes and tasks, and making decisions when selecting alternatives are examples of executive functions that operate in the background of thinking, language, and memory processes during text composition (Baddeley, 1996; Piolat et al., 2001). Readers with learning difficulties tend to perform lower on measures of the central executive system of working memory, indicating a lower ability to focus attention in a temporally flexible manner and to switch attention as needed (Swanson, 1999). The inability to shift attentional focus may lead to repetition of errors or persistence in repeating the same response (Lezak, 1995). In addition, studies confirm that individuals with dyslexia typically perform lower in organising, automatising, and integrating multiple processes, and perform lower on inhibition and shifting/cognitive flexibility tasks (e.g., Helland and Asbjørnsen, 2000; Berninger et al., 2006a,b; Altemeier et al., 2008). For example, there is already a large body of research showing that individuals with dyslexia have difficulty automatising reading skills but also other language related skills such as language fluency including naming speed and verbal retrieval (e.g., Denckla and Rudel, 1976; Yap and van der Leij, 1994; Berninger, 2001; Vellutino et al., 2004). The degree of automatization of a given skill has been tested by introducing a second task to be performed more or less concurrently with the first task. The performance on the dual-task is better if the individual is better able to automatise the second task. In their study, Hecht et al. (2004) compared performance in a single-task condition and a dual-task condition to test whether faster readers were more able to automatise a single cognitive task, which should subsequently help them perform better in the dual-task situation. The results showed that faster adult readers automatised the predictable cognitive task more quickly and performed better on the dual-task than slower readers. They suggested that slower readers need more practice to achieve optimal performance.

Such a dual-task method is widely used in cognitive psychology (e.g., Kellogg, 1994; Baddeley and Andrade, 2000), whereas the TT may be even more challenging for a fluent performance because it contains also a third requirement. Moreover, the second task is hardly predictable because the audio signal occurs in a variable interval schedule, while the third task is more predictable because it has only three options, i.e., three choices of metacognitive processes to be reported introspectively (planning, translating, or revising). Unpredictable and varied, unlike predictable and constant tasks, are harder to automatise (Shiffrin and Dumais, 1981; Hecht et al., 2004), which provides an opportunity to measure cognitive effort in the secondary task of TT. On the other hand, if the audio signals are predictable, i.e., if they occur in a certain rhythm, then reaction time does not represent cognitive effort, because participants can anticipate the audio signal and respond automatically (or rhythmically), probably even without paying attention to the audio signal. However, TT involves three tasks that must be performed fluently and (more or less) concurrently by flexibly switching focus between tasks and keeping maintaining attention on the writing task. TT performance in individuals with typical reading skills is expected to become more fluent and, to some extent, also more automatic with training, i.e., without difficulty in performing three tasks in the correct order (focusing on the first task and, after audio stimuli, performing the second and then third tasks), in learning the instructions for direct introspection of metacognitive processes in the third task, or in directing focus to the first task and not to the other two. Perhaps it is more challenging for adults with dyslexia to produce such a fluent performance than for those with typical reading skills, which could manifest as reactivity of TT in the dyslexia group. While previous studies have shown that such trained TT performance has no effect on the writing process and writing product of individuals with typical reading skills, at least not with known measures of writing process and writing product (e.g., Penningroth and Rosenberg, 1995; Ransdell, 1995; Piolat et al., 1996), it is not known if the same is true for individuals with dyslexia. There is a reasonable concern that adults with dyslexia may have difficulty performing TT fluently, which could be reflected in writing processes or text quality characteristics. Therefore, it is important to measure possible reactivity effects when TT is used with individuals with dyslexia.

To sum up, TT reactivity to writing processes or product has not been demonstrated in most previous studies with populations with typical language skills (e.g., Penningroth and Rosenberg, 1995; Piolat et al., 1996), with the exception of Wengelin et al. (2014), who found more keystrokes and more revisions in the condition without TT. However, TT reactivity has not yet been tested in populations with language disorders, such as individuals with dyslexia. There is also a lack of data obtained by combining TT with KSL tools to measure TT reactivity.

Therefore, the first goal of this study was to investigate whether the TT method affects writing processes and/or written product in adults with dyslexia compared to a control group of adults with typical reading skills. It might be expected that adults with dyslexia may have difficulty in performing concurrently three tasks fluently, which could be reflected on some writing processes characteristics or in the text quality. Although many studies have not found evidence of TT reactivity in typical readers, some have, and studies with readers with dyslexia are non-existent. The second goal was to investigate whether the TT method affects the writing process and/or written product independently of dyslexia. From previous studies, it appears that some process measures could be disrupted by TT, e.g., revisions. And the third goal is to investigate group differences, i.e., to compare adults with dyslexia and adults with typical reading skills in terms of text quality and writing process measures.

## 2. Method

### 2.1. Participants

Two groups of adult native Croatian speakers participated in this study: (1) 20 adults with developmental dyslexia (DYS), and (2) 20 adults with typical reading skills (TRS) as a control group.

All participants with dyslexia had a history of dyslexia without other comorbidities and were additionally evaluated by a speech-language pathologist and a psychologist for the purpose of this study. The inclusion of participants in the dyslexia or control group was confirmed using tasks considered to measure core deficits in dyslexia (e.g., Ramus, 2003; Vellutino et al., 2004; Ramus and Szenkovits, 2008): (a) *reading speed* and *accuracy* (words and pseudo-words), (b) *spoonerism*, (c) *rapid automatised naming* (RAN), and (d) *digit span task (forward and backward)*. According to the univariate t-tests, the performance of the groups differed significantly on all tasks performed, with adults with dyslexia scoring lower (see analysis in Supplementary Table S1). Participants with dyslexia showed strong significant developmental delays in reading fluency and accuracy and in phonological processing tasks. Additionally, the two groups, DYS and TRS, were matched by age (ranging from 18 to 38 years), gender (13 males and 7 females per group), educational level, and nonverbal cognitive ability. All participants achieved a score above-1.5 SD on the nonverbal test (Raven’s Progressive Matrices; Raven, 1994). In terms of educational level, 15–25% of participants in both groups had attained a lower level of education (14 years of education), 40–45% of participants in both groups were post-secondary students, and 20–30% of participants in both groups had attained a higher level of education (BA, MA or higher-level degree).

All participants have regular eyesight (with or without aids) and were frequent users of keyboards, i.e., they write daily using a keyboard (90% of all participants; 90% of the dyslexia group and 95% of TRS) or several times a week (10% of all participants; 10% of the dyslexia group and 5% of TRS).

Prior to participation, all participants were asked to read and sign an informed consent form. Ethical approval for this study was obtained from the research ethics committee of the Centre for Postgraduate Studies Language and Cognitive Neuroscience, University of Zagreb (643–02/14–02/12; 380–130/127–17-13).

### 2.2. Variables

#### 2.2.1. Product analysis – text quality

Text quality was assessed using text-based and reader-based measurements. Text-based measures were divided into three text quality measures: (1) productivity, (2) complexity, and (3) accuracy. (1) Productivity was measured as text length in terms of word count. (2) Complexity included measures of syntactic complexity (number and proportion of simple and complex sentences) and lexical complexity (mean word length, longer words are considered more complex because more time is required to retrieve their meaning and they tend to be more morphologically complex). (3) Accuracy was measured as the frequency of different types of errors, such as spelling, capitalization, punctuation, grammar, semantics, pragmatics, and others (see more in Table 1).

**Table 1:** Variables of product analysis.

Conceptual definition	Variable	Operational definition
Productivity	Text length	Number of words
		Number of sentences
Complexity	Syntactic complexity	Mean length of sentence
		Number of simple sentences
		Number of complex sentences
	Lexical complexity	Mean word length
Accuracy	Conventions	*Spelling* – application of *č/ć* and *ije/je* rules; omission of diacritical marks; grapheme omission (e.g., in the infinitive); substitution and addition; inappropriate use of short and long forms of prepositions: s/sa, k/ka
		*Capitalization –* at the beginning of the sentence, proper nouns, capitalizing words that should not be capitalized
		*Punctuation –* missing or incorrect punctuation at the end of a sentence, omission of a coma in a list, inappropriate punctuation mark in the middle of a sentence
	Grammar	Inappropriate inflection, copula omission
	Semantics	Inappropriate use of word meaning, inappropriate preposition use
	Pragmatics or other	Incomplete sentence, double word, missing word (e.g., preposition), incorrect word order
Text quality (reader-based)	Content	Relevance, thoroughness, persuasiveness, creativity - diversity of ideas, originality
	Coherence	Connection of ideas, fluidity of discourse
	Syntax	Sentence structure, congruence, variety of structures
	Vocabulary	Vocabulary richness and diversity, colloquial expressions

Three independent readers (students in the master’s program in Speech and language pathology) rated the texts on four reader-based measures on text quality (adapted from Alves et al., 2016): (1) content, (2) coherence, (3) syntax, and (4) vocabulary (see more in Table 1). They were instructed and trained on how to evaluate the text-quality using the four criteria and a 5-point scale. To test inter-rater reliability, the intraclass correlation coefficient (ICC) was calculated for each text-quality criterion and for the total score (sum of the four text-quality measures), between raters. The ICC reflects the degree of correlation and agreement between the measures (Koo and Li, 2016). ICC estimates and their 95% confidence intervals were calculated using the SPSS 26 statistical package (SPSS Inc., Chicago, IL) based on an average measure of absolute agreement (k3) and a 2-way random effects model. The ICCs were as follows: (1) texts without TT: content (0.68), coherence (0.61), syntax (0.55), vocabulary (0.50), and overall score (0.61); (2) texts with TT: content (0.59), coherence (0.55), syntax (0.50), vocabulary (0.53), and overall score (0.57). All ICC scores can be interpreted as moderate, ranging from 0.50 to 0.74 (Koo and Li, 2016). In studies with quality assessment of texts, a moderate ICC is usually obtained so it can be considered an “acceptable” score (e.g., Hootman et al., 2011; Lockspeiser et al., 2013; Baethge et al., 2019; Saudek et al., 2020). However, a low ICC could reflect lower measurement agreement, but could also reflect lack of variability among subjects, small number of subjects, or small number of raters (Koo and Li, 2016). In further analysis, the average scores for each text-quality measure and the total scores of the four text-quality measures for both conditions were used.

#### 2.2.2. Process analysis

Keystroke logging (KSL) is a method of recording writing using a keyboard. The entire writing session was recorded in both conditions (writing with and without TT) using Inputlog 7.0 (Leijten and Van Waes, 2013). Participants wrote their texts in the Word Office environment, with automatic spelling or grammar corrections disabled. The Croatian keyboard (with five additional special characters Č, Ć, Ž, Š, and Đ) with QWERTZ layout was used. Participants were allowed to use only keyboard and mouse, and to have only one Word Office window open. The *Inputlog 8.0* (Leijten and Van Waes, 2013) was used to analyse several parameters, including: (1) revisions, (2) typewriting, and (3) some product to process ratios (see Table 2 for more details; and Leijten and Van Waes, 2019). All data were extracted using three Inputlog analysis options: Summary and Revision.

**Table 2:** Variables of process analysis.

Variable	Operational definition
Total process time (s)	The time interval between starting and ending of logging (limited to 25 min)
Total active writing time (s)	Total time spent in writing, i.e., in keystroking without pause
Revisions	
Revisions – Insertions	Total number of insertions of paragraphs, sentences, or words
Revisions – Deletions	Total number of deletions of paragraphs, sentences, or words
Number of R-bursts	Total number of sequences of text production terminated by a revision or by a normal text production at the end of the text produced so far
Mean R-bursts time (s)	An average time of sequences of text production terminated by a revision or by a normal text production at the end of the text produced so far
Typewriting	
Total number of characters (incl. spaces)	Total number of characters produced in the writing process
Total number of characters (excl. spaces)	
Number of characters per min. (incl. spaces)	Total number of characters typed within document / length of the writing process in minutes
Number of characters per min. (excl. spaces)	
Total keystrokes	Total number of keystrokes incl. Inserted and replaced characters in the document
Total non-character keys	Total number of non-character keystrokes (i.e., backspace, arrow, delete) in the document
Total typed (incl. spaces)	Total number of keystrokes (without revisions)
Total typed (excl. spaces)	
Total typed per min. (incl. spaces)	Total number of keystrokes (without revisions) per minute
Total typed per min. (excl. spaces)	
Product/process	
Produced ratio (incl. spaces)	Total number of characters in the final text and the total number of non-character keys / total number of characters produced during the writing process
Characters (incl. spaces)	Total number of characters in the final text/the total number of characters typed during the writing process
Characters (excl. spaces)	
Words	Total number of words in the final text/total number of words during the writing process

### 2.3. Procedure

Each participant had to write two narrative texts elicited by two visually balanced drawings (balanced in terms of: number of characters and actions, context, and digital painting technique) in two conditions: (1) with TT, and (2) without the TT. This single structured stimulus method was used because such a stimulus has been shown to discriminate well between the performance of individuals with language disorders and the typical population (e.g., Dalton and Richardson, 2019). Furthermore, by controlling for the topic variable, such a method allows for a more accurate comparison of a range of linguistic and non-linguistic measures across participants at the discourse level. Moreover, the use of a single structured stimulus method can control, to some extent, long-term background base knowledge, i.e., knowledge about the topic that influences fluency and quality of writing (e.g., Flower and Hayes, 1980; Kellogg, 2008). (1) In the first condition, before writing the main text with the TT, participants were trained for about 15 min to learn the second and the third tasks of the TT. The training task was analogous to the experimental task. The triple task was performed using a computer, Word Office 2010 software, a keyboard, and a response box. In the triple task, participants performed three tasks more or less concurrently. The first task involved writing a text, the second task required the participant to respond as quickly as possible to simple audio stimuli (in this case, randomly every 15 to 45 s) by pressing the key on the response box with the nondominant hand, and the third task required the participant to report the current process by pressing the appropriate key on the response box (planning, translating, or revising). (2) The second condition required participants to write a narrative text without the TT. The two conditions (with TT vs. without TT) were randomized between participants to avoid serial effects; the elicitation materials were not randomized between the conditions.

The time allowed for writing the texts was limited to 25 min per text. Participants spent approximately the same amount of time writing both texts, i.e., total processing time did not differ significantly between the two conditions in either group [*F*(1,31) = 0.03, *p* = 0.859; see Table 3].

**Table 3:** Text quality: interaction effects (*F*, *p*) and descriptive data (*M*, SD).

					TRS		DYS	
					With TT	No TT	With TT	No TT
Text quality variables	*F*	*df1, df2*	*p*	BFexcl	M (SD)	M (SD)	M (SD)	M (SD)
Productivity (*n* of words)	0.07	1, 38	0.800	3.214	267.15 (92.626)	282.75 (112.370)	192.10 (97.882)	202.85 (122.895)
Productivity (*n* of sentences)	0.26	1, 38	0.612	7.712	17.80 (9.384)	18.20 (9.065)	13.05 (6.476)	12.70 (6.546)
Lexical complexity (mean word length)	2.45	1, 38	0.126	0.820	4.49 (0.241)	4.73 (0.238)	4.46 (0.221)	4.58 (0.238)
Syntactic complexity (average sentence length)	0.06	1, 38	0.814	9.913	17.05 (4.239)	17.41 (6.650)	15.68 (3.600)	16.35 (4.450)
Syntactic complexity (*n* of simple sentences)	0.21	1, 38	0.647	5.424	3.85 (4.146)	5.00 (6.139)	2.30 (2.618)	2.80 (3.968)
Syntactic complexity (proportion of simple sentences)	0.00	1, 38	0.961	9.225	0.19 (0.149)	0.22 (0.191)	0.14 (0.127)	0.17 (0.203)
Syntactic complexity (*n* of complex sentences)	0.01	1, 38	0.941	4.299	13.95 (6.386)	13.20 (6.014)	10.75 (4.387)	9.90 (4.436)
Syntactic complexity (proportion of complex sentences)	0.00	1, 38	0.961	9.397	0.81 (0.149)	0.78 (0.191)	0.86 (0.127)	0.83 (0.201)
Accuracy (*n* of errors)	2.54	1, 38	0.119	0.696	3.60 (3.331)	3.40 (3.515)	12.90 (7.326)	10.20 (8.483)
Mechanical errors	0.387	1, 38	0.538	3.515	3.20 (2.966)	3.35 (3.483)	10.95 (6.985)	10.10 (8.416)
Grammatical errors	0.765	1, 38	0.387	2.365	0.10 (0.308)	0.15 (0.366)	1.25 (1.618)	0.90 (0.970)
Text-quality (reader-based)	1.941	1, 38	0.172	1.806	12.00 (3.877)	12.35 (3.535)	10.05 (2.443)	8.92 (3.228)
Content	2.064	1, 38	0.159	2.465	2.98 (1.062)	3.12 (1.016)	2.60 (0.769)	2.27 (0.940)
Coherence	2.107	1, 38	0.155	2.665	3.05 (1.125)	3.20 (1.034)	2.72 (0.744)	2.42 (0.904)
Syntax	0.298	1, 38	0.588	3.864	2.98 (0.958)	2.98 (0.895)	2.35 (0.679)	2.183 (0.848)
Vocabulary	0.800	1, 38	0.140	1.276	2.98 (0.958)	3.05 (0.789)	2.38 (0.575)	2.05 (0.711)

Prior to the TT, baseline reaction time (RT) was measured as the average time between the moment the participant heard a simple audio stimulus and the moment he/she pressed the red button with the nondominant hand on the response box over 50 trials. The simple audio stimulus was given randomly at time intervals between 5 and 15 s. The task consists of 55 trials in total, with the first five trials being for practice and therefore not included in the calculation of the baseline RT. Participants had to keep both hands on the keyboard the entire time while waiting for the audio stimulus, as this simulated the recording of reaction time as in TT. This ensures the possibility of comparing two measured reaction times (secondary and simple RT). According to the results of the t-test, DYS and TRS group does not differ significantly in the baseline reaction time, i.e., reaction to the simple audio stimuli (*t* = −1.89; df = 38; *p* = 0.066; MTRS = 673.71 (SD = 124.437), MDYS = 749.76 (SD = 129.493)); the one that is subtracted from the RT in the TT condition. For a comparison, in the TT condition (secondary task) participants had longer RT where an average for TRS group is MTRS = 1180.18 (SD = 216.107) and for dyslexia group is MDYS = 1460.93 (SD = 443.434). In addition, a similar method of recording RT by pressing button has been used in previous studies that also focused on measuring writing processes (e.g., Piolat et al., 2005; Alamargot et al., 2006; Beauvais et al., 2011; Limpo, 2018; Limpo and Alves, 2018).

## 3. Results

To examine whether the triple task had a differential effect on text quality between groups of adults with dyslexia and TRS adults, mixed ANOVAs were conducted, using IBM SPSS Statistics 26 (IBM Corp, 2019). Group membership was a between-subjects factor, whereas measures of writing processes and text quality for two different texts (one with and one without TT) were within-subjects’ factors. The interaction effects of group (dyslexia vs. TRS) and TT condition (with vs. without) were tested, as well as the main effects of TT condition and group on measures of writing processes and text quality. The first main effect shows differences between two TT conditions independent of group, the second differences between two groups, independent of TT condition, in text quality and writing process variables.

Comparing to frequentists approach, Bayes statistics provide a coherent method to determining whether non-significant results support a null hypothesis over a theory, or whether the data are just insensitive (Dienes, 2014). Frequentist and Bayesian methods have different assumptions about the data and model parameters. Frequentist statistics assume that the null hypothesis is true and calculate the probability of obtaining the observed data, whereas Bayesian statistics incorporate prior assumptions and update them based on the observed data, providing evidence either in favour of or against the null hypothesis. While *p*-values in frequentist analyses can only reject the null hypothesis, the Bayes factor can indicate evidence for the null hypothesis (and alternative), allowing for confirmation of the hypotheses, which is very informative when trying to confirm the null hypothesis as in the current research, i.e., when testing interaction effects. Incorporating both approaches into a study allows researchers to explicitly address these assumptions, which increases the rigor of the study and allows readers to assess the strength and reliability of the conclusions (Kelter, 2020). So, the Bayesian statistical mixed ANOVAs were implemented as a supplement to null hypothesis significance tests, using JASP 0.17.1 (JASP Team, 2023). The models were compared to the null model (which included the subject variable and random slopes) in each case and calculated the BF_excl_ values across all models, using its default prior. BF_excls_ reflect how much more likely it is that the effect does not exist (H0) compared to that it does (H1), given the data. BF_excl_ values above 1 indicate evidence in favour of H0 over the alternative hypothesis; BFexcl values below 1 indicate evidence in favour of the alternative hypothesis; while values close to 1 indicate that the data are equally likely for null and the alternative hypothesis. Higher values indicate stronger evidence; values of 1–3 indicate weak evidence, 3–10 indicate moderate evidence, and above 10 indicate strong evidence (Jeffreys, 1961).

### 3.1. Text quality – interactions and main effects

The necessary assumptions for conducting mixed ANOVAs were largely met. The dependent variables are normally or approximately normally distributed within the two subgroups. Homogeneity of variances tested with Box’s *M*-test was assumed for all variables except Syntactic complexity and those related to Accuracy. Therefore, the Greenhouse-Geiser corrected degrees of freedom were used to evaluate the significance of the corresponding F for those variables. The results obtained showed no significant interaction between group membership and writing texts with or without a triple task in all measured dimensions of text quality (text-based and reader-based; see Table 4). Indeed, the quality of texts written with or without the triple task did not differ significantly between adults with dyslexia or TRS adults.

**Table 4:** Writing process: interaction effects (*F*, *p*) and descriptive data (*M*, SD).

					TRS		DYS	
					With TT	No TT	With TT	No TT
Writing process variables	*F*	df1, df2	*p*	BFexcl	*M* (SD)	*M* (SD)	*M* (SD)	*M* (SD)
Total process time (s)	0.03	1, 31	0.859	10.394	899.08 (233.537)	882.74 (200.995)	849.43 (269.717)	818.63 (286.210)
Total Active writing time (s)	1.80	1, 29	0.191	2.459	235.94 (73.533)	220.39 (68.302)	158.57 (65.361)	170.69 (95.577)
Revisions								
Revisions – Insertions	0.11	1, 29	0.917	2.776	34.18 (30.338)	7.12 (9.440)	38.43 (36.712)	10.07 (13.141)
Revisions – Deletions	0.49	1, 31	0.619	2.013	91.76 (30.862)	65.12 (22.355)	83.21 (29.569)	59.64 (27.709)
Number of R-bursts	0.00	1, 24	0.960	4.191	64.00 (33.512)	50.86 (25.270)	57.167 (32.330)	44.75 (21.201)
Mean R-bursts time (s)	0.04	1, 26	0.850	2.093	6.85 (2.063)	9.06 (4.772)	7.40 (2.378)	9.42 (3.577)
Typewriting								
Total number of characters (incl. spaces)	0.53	1, 35	0.472	1.899	1533.16 (479.369)	1666.05 (574.321)	1106.722 (548.443)	1157.56 (675.881)
Number of characters per min. (incl. spaces)	2.37	1, 30	0.134	0.510	100.75 (18.903)	113.85 (24.096)	75.51 (26.723)	78.46 (30.982)
Total number of characters (excl. spaces)	0.80	1, 34	0.379	1.393	1274.06 (396.785)	1399.11 (470.880)	953.94 (447.500)	946.33 (550.014)
Number of characters per min. (excl. spaces)	2.62	1, 30	0.116	0.416	82.68 (15.418)	93.79 (19.926)	61.85 (21.950)	64.14 (25.186)
Total keystrokes	0.05	1, 35	0.821	4.231	2071.58 (1040.090)	2010.84 (603.464)	1489.00 (698.520)	1480.17 (691.794)
Total non-character keys	1.01	1, 35	0.323	1.071	39.37 (27.603)	40.42 (29.624)	27.28 (17.963)	23.72 (29.624)
Total typed (incl. spaces)	0.52	1, 35	0.478	2.262	1848.68 (8541.105)	1964.37 (591.294)	1386.28 (564.767)	1419.11 (684.219)
Total typed per min. (incl. spaces)	3.77	1, 30	0.062	0.278	121.92 (19.539)	134.65 (19.695)	95.56 (27.660)	97.46 (30.210)
Total typed (excl. spaces)	0.58	1, 35	0.451	1.656	1501.79 (436.502)	1620.79 (483.959)	1114.44 (457.594)	1161.50 (558.451)
Total typed per min. (excl. spaces)	3.99	1, 30	0.055	0.215	98.97 (15.819)	111.01 (16.477)	76.875 (22.772)	79.78 (24.897)
Product/process								
Produced ratio (incl. spaces)	0.40	1, 35	0.531	4.706	0.80 (0.119)	0.84 (0.084)	0.77 (0.084)	0.78 (0.144)
Characters (incl. spaces)	0.01	1, 35	0.919	4.845	0.83 (0.060)	0.84 (0.077)	0.79 (0.103)	0.83 (0.060)
Characters (excl. spaces)	0.10	1, 35	0.749	9.571	0.84 (0.060)	0.84 (0.077)	0.80 (0.101)	0.80 (0.118)
Words	0.06	1, 35	0.802	1.278	0.75 (0.090)	0.87 (0.078)	0.75 (0.090)	0.81 (0.057)

Bayesian mixed ANOVA analysis shows that most interaction effects (group x condition) for the text quality variables are BF_excl_ > 1 and in favour of null hypothesis, i.e., in the range BF_excl_ = 1.276 to 9.397; while the interaction effects for lexical complexity (BF_excl_ = 0.820) and accuracy (n of errors) (BF_excl_ = 0.696) are slightly below 1, suggesting that there is not enough evidence to support null or alternative hypothesis. Five variables show weak evidence in favour of null hypothesis, with BF_excl_ mostly between 2 and 3, and nine variables show moderate evidence (see all BF_excl_s in Table 4). Detailed Model Comparisons and Analysis of Effects are in Supplementary Tables S2, S3. The Bayesian analysis suggests that further research should be conducted on the reactivity of TT for text quality variables and, in particular, for seven variables with low evidence and two with no evidence for the null hypothesis.

The main effect of the TT condition for the lexical complexity (mean word length) was significant [*F*(1,38) = 22.36, *p* = 0.000, η_p_^2^ = 0.370; BF_excl_ = 0.002] implying that words are longer in the condition without the TT (see descriptive statistics in Table 4). Other main effects of the TT condition were not significant.

The main effect of group was significant for number of words [*F*(1,38) = 5.68, *p* = 0.022, η_p_^2^ = 0.130, BF_excl_ = 0.478], number of sentences [*F*(1,38) = 4.50, *p* = 0.040, η_p_^2^ = 0.106, BF_excl_ = 0.746], average sentence length [*F*(1,38) = 4.33, *p* = 0.044, η_p_^2^ = 0.102, BF_excl_ = 2.633], total number of errors [*F*(1,38) = 20.82, *p* = 0.000, η_p_^2^ = 0.354, BF_excl_ = 0.003], number of mechanical errors [*F*(1,38) = 18.33, *p* = 0.000, η_p_^2^ = 0.325, BF_excl_ = 0.008], number of grammatical errors [*F*(1,38) = 21.334, *p* = 0.000, η_p_^2^ = 0.360, BF_excl_ = 0.0015], total text-quality (reader-based) [*F*(1,38) = 8.90, *p* = 0.005, η_p_^2^ = 0.190, BF_excl_ = 0.145], and all text-quality measures: content [*F*(1,38) = 5.89, *p* = 0.020, η_p_^2^ = 0.134, BF_excl_ = 0.417], coherence [*F*(1,38) = 4.54, *p* = 0.040, η_p_^2^ = 0.107, BF_excl_ = 0.709], syntax [*F*(1, 38) = 10.45, *p* = 0.003, η_p_^2^ = 0.216, BF_excl_ = 0.096], vocabulary [*F*(1,38) = 15.31, *p* = 0.000, η_p_^2^ = 0.287, BF_excl_ = 0.017]. When compared with TRS adults, adults with dyslexia in their texts produced less words and sentences; they made more mechanical and grammatical errors; and the overall text-quality was poorer including content, coherence, syntax, and vocabulary (see descriptive statistics in Table 4).

### 3.2. Writing processes – interactions and main effects

The necessary assumptions for conducting mixed ANOVAs for the group of writing process variables were tested as in the previous analysis, and they were largely satisfied. The dependent variables are normally or approximately normally distributed within the two subgroups. Homogeneity of variances tested with Box’s M-test was assumed for all variables except for the Total keystrokes, so Greenhouse-Geiser corrected degrees were used in the related analyses.

The results showed no significant interaction between group membership and writing in texts with or without a triple task for all measured dimensions of the writing process (see Table 3). Like the previous case, the measured writing processes in texts written with or without the triple task did not differ significantly between adults with dyslexia or TRS adults.

According to Bayesian mixed ANOVA, most of interaction effects (group x condition) for writing process variables are BF_excl_ > 1 and in favour of null hypothesis, i.e., in the range from BF_excl_ = 1.071 to 10.394; while interaction effects for number of characters per min. (incl. spaces) (BF_excl_ = 0.510), number of characters per min. (excl. spaces) (BF_excl_ = 0.416) and total typed per min. (excl. spaces) (BF_excl_ = 0.215) are below 1. Ten variables show weak evidence for null hypothesis, and eight variables show moderate evidence (see all BF_excl_ in Table 3). Detailed Model Comparisons and Analysis of Effects are in Supplementary Tables S2, S3. The Bayesian analysis implies that further research on the reactivity of TT should be conducted for text writing process variables, at least for ten variables with low evidence and four variables that lack evidence for the null hypothesis.

Some of the main effects of the TT condition for the process variables cannot be interpreted validly (e.g., revisions) because they interfere with a technical aspect of the triple task implementation, i.e., the response box was the keyboard extension, and the strokes were logged as deletions or insertions. However, some main effects of the TT condition for the process variables do not interfere to this technical aspect, so they can be interpreted; and those that were found to be significant indicate the reactivity of TT regardless of group. In the typewriting analysis, the total number of characters per minute including spaces [*F*(1,30) = 5.931, *p* = 0.021, η_p_^2^ = 0.165, BF_excl_ = 1.468] and excluding spaces [*F*(1,30) = 6.054, *p* = 0.020, η_p_^2^ = 0.168, BF_excl_ = 1.321] was higher in the condition without TT; similarly, more keys were typed per minute including spaces [*F*(1,30) = 6.863, *p* = 0.014, η_p_^2^ = 0.186, BF_excl_ = 0.253] and excluding spaces [*F*(1,30) = 10.647, *p* = 0.003, η_p_^2^ = 0.262, BF_excl_ = 0.077] in the condition without TT. The ratio between the total number of words in the final text and the total number of words produced during the writing process was higher in the condition without TT [*F*(1,35) = 36.901, *p* = 0.000, η_p_^2^ = 0.457, BF_excl_ = 3.410 × 10^−4^] implying that more words were deleted in the condition with TT. For the direction of the differences see descriptive statistics in the Table 3.

The main effect of group was significant for total active writing time [*F*(1,29) = 6.23, *p* = 0.018, η_p_^2^ = 0.177, BF_excl_ = 0.394], total number of characters (incl. spaces) [*F*(1,34) = 7.57, *p* = 0.009, η_p_^2^ = 0.182, BF_excl_=], total number of characters (excl. spaces) [*F*(1,30) = 12.98, *p* = 0.001, η_p_^2^ = 0.302, BF_excl_ = 0.262], number of characters per min. (incl. spaces) [*F*(1,30) = 12.98, *p* = 0.001, η_p_^2^ = 0.302, BF_excl_ = 0.038], number of characters per min. (excl. spaces) [*F*(1,30) = 13.47, *p* = 0.001, η_p_^2^ = 0.310, BF_excl_ = 0.031], total keystrokes [*F*(1,35) = 5.88, *p* = 0.021, η_p_^2^ = 0.144, BF_excl_=], total typed (incl. spaces) (F(1,35) = 7.21, *p* = 0.011, η_p_^2^ = 0.171, BF_excl_ = 0.454), total typed per min. (incl. spaces) [*F*(1,30) = 14.70, p = 0.001, η_p_^2^ = 0.329, BF_excl_ = 0.020], total typed (excl. spaces) [*F*(1,35) = 7.70, p = 0.009, η_p_^2^ = 0.180, BF_excl_ = 0.261], total typed per min. (excl. spaces) [*F*(1,30) = 15.24, p = 0.000, η_p_^2^ = 0.337, BF_excl_ = 0.016], and proportion of number of words in final text and during the writing process [*F*(1,35) = 15.24, *p* = 0.049, η_p_^2^ = 0.106, BF_excl_ = 0.713]. When compared with TRS adults, adults with dyslexia spent less time in active writing, produced a smaller number of characters and characters per min., used less keystrokes, less typed in total and per min., and had a less words in the final text comparing to the number of words produced during the writing process (see descriptive statistics in Table 3).

## 4. Discussion

The purpose of this study was to investigate the reactivity of the triple task method in adults with dyslexia and to compare them with a control group of adults with typical reading skills. It was expected that adults with dyslexia would have difficulty performing the triple task, which could be reflected in writing processes or text quality characteristics. Most previous studies have not confirmed TT reactivity to writing processes or written product in population with typical development (e.g., Penningroth and Rosenberg, 1995; Ransdell, 1995; Piolat et al., 1996) that is, only Wengelin et al. (2014) found more keystrokes and more revisions in the condition without TT. On the other hand, TT reactivity has not been tested in individuals with language disorders such as dyslexia. Moreover, there are no data on TT reactivity measured with KSL measures.

The results of the present study showed no significant interaction effect between group membership and writing condition, suggesting that writing processes and text quality in the group of adults with dyslexia and in the control group are not differentially affected by TT. Furthermore, the fact that no single statistically significant effect was found at *p* < 0.05 across many comparisons strengthens the evidence that there is no differential reactivity (because multiple comparisons increase the probability that *p* < 0.05 occurs by chance in one of these comparisons). Thus, with regard to the first objective of this study, it can be assumed that the use of the triple task in adults with dyslexia compared to adults with typical reading skills probably has no effect on the writing processes or product variables measured in this study (as shown in Tables 3 4). It appears that the triple task is a method that is stable enough not to be affected by difficulties that lie in the background of dyslexia and the training of the TT was sufficient to learn the steps of the TT, so that the whole task did not represent a greater additional burden for adults with dyslexia than for the comparison group. However, the Bayesian analyses showed that the evidence for the null hypothesis is rather weak for some interactions, so further research is needed to empower those findings. Also, further research may reveal how reactive the TT is for individuals with dyslexia who write more complex texts and discourse. It is possible that the TT is more responsive to writing processes for more complex texts. For example, Janssen et al. (1996) found that thinking aloud protocols influenced more complex writing processes than simple writing processes (planning level and pause length).

Regarding the second objective, TT reactivity was tested independently of group membership on writing processes and text quality, i.e., TT reactivity was tested independently of dyslexia. The results showed some effects of reactivity. In the condition without TT, the total number of characters per minute was higher and more keys were typed per minute, while the ratio between the total number of words in the final text and the total number of words produced during the writing process showed that more words were deleted in the condition with TT. Wengelin et al. (2014) found that more keystrokes and more revisions were made without TT. Thus, along with our study, they also showed that the triple task may be reactive to some writing processes.

For the third objective, a group of adults with dyslexia was compared to adults with typical reading skills in terms of text quality and writing process. The current study confirmed that adults with dyslexia produced fewer words and sentences in their texts; made significantly more mechanical and grammatical errors when composing texts; produced texts of lower quality in terms of content, coherence, syntax, and vocabulary. Process analysis revealed that adults with dyslexia spent less time actively writing, took more breaks compared to writing time, produced fewer characters per minute, indicating lower transcribing fluency; used fewer keystrokes, had fewer words in the final text compared to the text produced, and typed less. Similar results were found in some previous studies showing that adults with dyslexia produce texts of generally poorer quality and at lexical, structural, and thematic levels compared to controls (Sterling et al., 1998; Connelly et al., 2006; Wengelin, 2007; Torrance et al., 2016). They produce more morphosyntactic, spelling, memory-related, punctuation, and capitalization errors (Sterling et al., 1998; Connelly et al., 2006; Tops et al., 2013; Torrance et al., 2016). Their essays are shorter (Sterling et al., 1998) and their handwriting is less fluent (Connelly et al., 2006).

Finally, some limitations of the study should be discussed. The first limitation stems from the small number of participants, resulting in a lower power of the study. A similar limitation is characteristic of most of the studies presented above. Although dyslexia is not that uncommon, with a prevalence of 5 to 12% in Europe (Dyslexia Compass, 2022), recruiting adults with dyslexia for research is complicated by the fact that this group is no longer in a regular school or health care system and therefore not as easily accessible. In addition, people with dyslexia may even avoid participating in research activities such as reading and writing because they do not want to be exposed to activities that will overwhelm them and make them feel uncomfortable. The second limitation refers to the procedure in which the conditions (TT and no TT) were randomized and not counterbalanced, i.e., the order of the conditions was randomly (not systematically) assigned to the participants. Randomization makes all possibilities equal, thus marginalizing potential serial effects, whereas systematic counterbalancing provides even better control for this type of bias. In addition, picture material was not randomized between conditions, i.e., in one condition, writing text was always elicited by the same prompt. However, the drawings were visually equalized in terms of number of characters and actions, context, and digital painting technique, making it highly unlikely that text quality was related to the properties of the prompt material. Furthermore, the results showed no differences in text quality between the two conditions in either group. Nevertheless, this limitation should be mentioned because randomization of the prompts could eliminate this potential bias. The third potential limitation or problem that should be considered in further research is the fact that participants had to write a narrative essay prompted by pictures. This is a task that could be considered mildly to moderately cognitively demanding. Perhaps a more cognitively demanding discourse, such as an expository text on a particular topic, would reveal more TT reactivity or differences between the groups studied.

## 5. Conclusion

When applying a new and complex writing method to a population that has difficulty reading and writing, researchers must be aware of the potential reactivity of the methods used. For example, it is still an open question whether individuals with dyslexia or other language disorders such as Aphasia (MA80.0, ICD-11, WHO, 2019/2021), Developmental language disorder (6A01.2, ICD-11, WHO, 2019/2021) are more sensitive to the TT method than individuals with typical reading or language skills, increasing the likelihood that the TT will be reactive. The results of the current study suggest that TT is unlikely to have differential effects on the writing process or written product in adults with dyslexia compared to adults with typical reading skills. However, further research is needed to strengthen these findings. Nevertheless, the current study and a similar study by Wengelin et al. (2014) provide evidence that the triple task may be reactive in some writing processes regardless of dyslexia. The current study showed that in the no TT condition, the total number of characters per minute was higher, more keys were typed per minute and more words were deleted. When comparing adults with dyslexia and adults with typical reading skills, it was found that adults with dyslexia produced fewer words and sentences in their texts, made more mechanical and grammatical errors, produced texts of lower overall quality, produced fewer characters per minute, used fewer keystrokes, typed less, and had fewer words in the final text than were produced. In addition, researchers need to pay careful attention to TT reactivity, especially in the area of reading and writing disorders, but also when the writing task (or discourse) is strenuous or when new measures of writing process or written product are used in research with TT.

## Data availability statement

The raw data supporting the conclusions of this article will be made available by the authors, without undue reservation.

## Ethics statement

The studies involving humans were approved by the Centre for Postgraduate Studies Language and Cognitive Neuroscience, University of Zagreb (643–02/14–02/12; 380–130/127–17-13). The studies were conducted in accordance with the local legislation and institutional requirements. The participants provided their written informed consent to participate in this study.

## Author contributions

MOT contributed significantly to the concept and design of the work, as it is part of her unpublished doctoral dissertation. She conducted the research, but also analyzed, interpreted, and discussed the data. JKK and RA critically reviewed the work and enriched it with their extensive knowledge and experience in writing articles. They were involved in the entire process of conducting the research and writing the paper. As supervisors, they guided and advised the first author through the extensive research process. All authors contributed to the article and approved the submitted version.
