# Supplementary material for: Reactivity of the triple task on writing processes and product in adults with dyslexia
Source: Front Psychol. 2023 Oct 9;14:1112274. doi: 10.3389/fpsyg.2023.1112274 (PMC10591105; doi:10.3389/fpsyg.2023.1112274)
Supplement: Supplementary file 1 [file Data_Sheet_1.docx]

Supplementary Material

*Supplementary Table 1: Tasks discriminative for dyslexia: Descriptive data and t-test*

| Variable | Group | *M* | *SD* | *Min.* | *Max.* | *t* | *df* | *p* | *Cohen’s d* |
| --- | --- | --- | --- | --- | --- | --- | --- | --- | --- |
| Word reading (A) | TRS | 109.70 | .57 | 108.00 | 110.70 | 5.09 | 38 | .000 | 1.65 |
|  | DYS | 105.25 | 3.86 | 97.00 | 110.00 |  |  |  |  |
| Word reading (S) | TRS | 62.15 | 17.42 | 36.00 | 118.00 | -5.15 | 38 | .000 | 1.67 |
|  | DYS | 112.25 | 39.87 | 66.00 | 197.00 |  |  |  |  |
| Pseudo-word reading (A) | TRS | 20.75 | .64 | 19.00 | 21.00 | 3.95 | 37 | .000 | 1.30 |
|  | DYS | 18.79 | 2.07 | 15.00 | 21.00 |  |  |  |  |
| Pseudo-word reading (S) | TRS | 14.85 | 3.59 | 10.00 | 24.00 | -6.77 | 37 | .000 | 2.23 |
|  | DYS | 32.37 | 10.73 | 18.00 | 55.00 |  |  |  |  |
| Spoonerism (A) | TRS | 13.85 | 2.52 | 6.00 | 16.00 | 3.24 | 38 | .002 | 1.05 |
|  | DYS | 10.00 | 4.68 | 1.00 | 16.00 |  |  |  |  |
| Spoonerism (S) | TRS | 132.58 | 46.68 | 65.00 | 222.00 | -7.08 | 33 | .000 | 2.46 |
|  | DYS | 294.94 | 86.25 | 205.00 | 520.00 |  |  |  |  |
| RAN (A) | TRS | 49.95 | .22 | 49.00 | 50.00 | 2.55 | 38 | .019 | 0.83 |
|  | DYS | 49.40 | .94 | 47.00 | 50.00 |  |  |  |  |
| RAN (S) | TRS | 24.95 | 4.24 | 20.00 | 36.00 | -3.38 | 38 | .002 | 1.10 |
|  | DYS | 31.15 | 7.01 | 20.00 | 44.00 |  |  |  |  |
| DS – forward | TRS | 7.00 | 1.08 | 5.00 | 9.00 | 3.71 | 38 | .001 | 1.20 |
|  | DYS | 5.85 | .88 | 5.00 | 8.00 |  |  |  |  |
| DS – backward | TRS | 5.80 | .95 | 3.00 | 9.00 | 4.61 | 38 | .000 | 1.50 |
|  | DYS | 4.25 | 1.16 | 3.00 | 8.00 |  |  |  |  |
| *TLS - typical reading skills; DYS – dyslexia; A – accuracy; S – speed; DS – digit span* | | | | | | | | | |

*Supplementary Table 2: Text quality: Bayesian mixed ANOVA - Model Comparison*

| **Model Comparison** | | | | | | | | | | | |
| --- | --- | --- | --- | --- | --- | --- | --- | --- | --- | --- | --- |
| **Productivity (n of words)** | | | | | | | | | | | |
| **Models** | | **P(M)** | | **P(M\|data)** | | **BF_M_** | | **BF_01_** | | **error %** | |
| Null model (incl. subject and random slopes) |  | 0.200 |  | 0.159 |  | 0.756 |  | 1.000 |  |  |  |
| group |  | 0.200 |  | 0.456 |  | 3.347 |  | 0.349 |  | 15.068 |  |
| RM Factor 1 + group |  | 0.200 |  | 0.231 |  | 1.199 |  | 0.689 |  | 2.599 |  |
| RM Factor 1 |  | 0.200 |  | 0.083 |  | 0.361 |  | 1.923 |  | 1.096 |  |
| RM Factor 1 + group + RM Factor 1 ✻ group |  | 0.200 |  | 0.072 |  | 0.311 |  | 2.203 |  | 16.253 |  |
|  | | | | | | | | | | | |
|  | | | | | | | | | | | |
| **Productivity (n of sentences)** | | | | | | | | | | | |
| **Models** | | **P(M)** | | **P(M\|data)** | | **BF_M_** | | **BF_01_** | | **error %** | |
| Null model (incl. subject and random slopes) |  | 0.200 |  | 0.271 |  | 1.485 |  | 1.000 |  |  |  |
| group |  | 0.200 |  | 0.531 |  | 4.536 |  | 0.510 |  | 7.254 |  |
| RM Factor 1 + group |  | 0.200 |  | 0.105 |  | 0.470 |  | 2.576 |  | 3.567 |  |
| RM Factor 1 |  | 0.200 |  | 0.061 |  | 0.262 |  | 4.411 |  | 0.840 |  |
| RM Factor 1 + group + RM Factor 1 ✻ group |  | 0.200 |  | 0.031 |  | 0.130 |  | 8.622 |  | 2.976 |  |
|  | | | | | | | | | | | |
|  | | | | | | | | | | | |
| **Lexical complexity (mean word length)** | | | | | | | | | | | |
| **Models** | | **P(M)** | | **P(M\|data)** | | **BF_M_** | | **BF_01_** | | **error %** | |
| Null model (incl. subject and random slopes) |  | 0.200 |  | 8.165×10^-4^ |  | 0.003 |  | 1.000 |  |  |  |
| RM Factor 1 |  | 0.200 |  | 0.459 |  | 3.398 |  | 0.002 |  | 0.888 |  |
| RM Factor 1 + group |  | 0.200 |  | 0.306 |  | 1.761 |  | 0.003 |  | 2.288 |  |
| RM Factor 1 + group + RM Factor 1 ✻ group |  | 0.200 |  | 0.234 |  | 1.220 |  | 0.003 |  | 1.932 |  |
| group |  | 0.200 |  | 4.776×10^-4^ |  | 0.002 |  | 1.709 |  | 1.089 |  |
|  | | | | | | | | | | | |

| **Syntactic complexity (average sentence length)** | | | | | | | | | | | |
| --- | --- | --- | --- | --- | --- | --- | --- | --- | --- | --- | --- |
| **Models** | | **P(M)** | | **P(M\|data)** | | **BF_M_** | | **BF_01_** | | **error %** | |
| Null model (incl. subject and random slopes) |  | 0.200 |  | 0.492 |  | 3.880 |  | 1.000 |  |  |  |
| group |  | 0.200 |  | 0.260 |  | 1.409 |  | 1.890 |  | 0.569 |  |
| RM Factor 1 |  | 0.200 |  | 0.145 |  | 0.676 |  | 3.404 |  | 1.095 |  |
| RM Factor 1 + group |  | 0.200 |  | 0.078 |  | 0.338 |  | 6.322 |  | 1.153 |  |
| RM Factor 1 + group + RM Factor 1 ✻ group |  | 0.200 |  | 0.025 |  | 0.101 |  | 20.017 |  | 2.310 |  |
|  | | | | | | | | | | | |

| **Syntactic complexity (n of simple sentences)** | | | | | | | | | | | |
| --- | --- | --- | --- | --- | --- | --- | --- | --- | --- | --- | --- |
| **Models** | | **P(M)** | | **P(M\|data)** | | **BF_M_** | | **BF_01_** | | **error %** | |
| Null model (incl. subject and random slopes) |  | 0.200 |  | 0.363 |  | 2.280 |  | 1.000 |  |  |  |
| group |  | 0.200 |  | 0.311 |  | 1.806 |  | 1.167 |  | 0.806 |  |
| RM Factor 1 |  | 0.200 |  | 0.149 |  | 0.701 |  | 2.434 |  | 1.092 |  |
| RM Factor 1 + group |  | 0.200 |  | 0.133 |  | 0.612 |  | 2.738 |  | 2.012 |  |
| RM Factor 1 + group + RM Factor 1 ✻ group |  | 0.200 |  | 0.044 |  | 0.184 |  | 8.241 |  | 4.654 |  |
|  | | | | | | | | | | | |

| **Syntactic complexity (proportion of simple sentences)** | | | | | | | | | | | |
| --- | --- | --- | --- | --- | --- | --- | --- | --- | --- | --- | --- |
| **Models** | | **P(M)** | | **P(M\|data)** | | **BF_M_** | | **BF_01_** | | **error %** | |
| Null model (incl. subject and random slopes) |  | 0.200 |  | 0.486 |  | 3.783 |  | 1.000 |  |  |  |
| group |  | 0.200 |  | 0.239 |  | 1.253 |  | 2.037 |  | 1.042 |  |
| RM Factor 1 |  | 0.200 |  | 0.166 |  | 0.797 |  | 2.926 |  | 1.243 |  |
| RM Factor 1 + group |  | 0.200 |  | 0.083 |  | 0.361 |  | 5.869 |  | 2.793 |  |
| RM Factor 1 + group + RM Factor 1 ✻ group |  | 0.200 |  | 0.026 |  | 0.108 |  | 18.422 |  | 2.641 |  |
|  | | | | | | | | | | | |

| **Syntactic complexity (n of complex sentences)** | | | | | | | | | | | |
| --- | --- | --- | --- | --- | --- | --- | --- | --- | --- | --- | --- |
| **Models** | | **P(M)** | | **P(M\|data)** | | **BF_M_** | | **BF_01_** | | **error %** | |
| Null model (incl. subject and random slopes) |  | 0.200 |  | 0.235 |  | 1.227 |  | 1.000 |  |  |  |
| group |  | 0.200 |  | 0.421 |  | 2.904 |  | 0.558 |  | 1.549 |  |
| RM Factor 1 + group |  | 0.200 |  | 0.192 |  | 0.953 |  | 1.220 |  | 5.004 |  |
| RM Factor 1 |  | 0.200 |  | 0.097 |  | 0.431 |  | 2.412 |  | 1.096 |  |
| RM Factor 1 + group + RM Factor 1 ✻ group |  | 0.200 |  | 0.055 |  | 0.233 |  | 4.270 |  | 3.564 |  |
|  | | | | | | | | | | | |

| **Syntactic complexity (proportion of complex sentences)** | | | | | | | | | | | |
| --- | --- | --- | --- | --- | --- | --- | --- | --- | --- | --- | --- |
| **Models** | | **P(M)** | | **P(M\|data)** | | **BF_M_** | | **BF_01_** | | **error %** | |
| Null model (incl. subject and random slopes) |  | 0.200 |  | 0.485 |  | 3.771 |  | 1.000 |  |  |  |
| group |  | 0.200 |  | 0.244 |  | 1.288 |  | 1.992 |  | 1.823 |  |
| RM Factor 1 |  | 0.200 |  | 0.164 |  | 0.785 |  | 2.957 |  | 0.928 |  |
| RM Factor 1 + group |  | 0.200 |  | 0.081 |  | 0.353 |  | 5.983 |  | 1.391 |  |
| RM Factor 1 + group + RM Factor 1 ✻ group |  | 0.200 |  | 0.026 |  | 0.106 |  | 18.725 |  | 3.424 |  |
|  | | | | | | | | | | | |

| **Accuracy (n of errors)** | | | | | | | | | | | |
| --- | --- | --- | --- | --- | --- | --- | --- | --- | --- | --- | --- |
| **Models** | | **P(M)** | | **P(M\|data)** | | **BF_M_** | | **BF_01_** | | **error %** | |
| Null model (incl. subject and random slopes) |  | 0.200 |  | 0.001 |  | 0.004 |  | 1.000 |  |  |  |
| group |  | 0.200 |  | 0.370 |  | 2.348 |  | 0.003 |  | 1.104 |  |
| RM Factor 1 + group |  | 0.200 |  | 0.364 |  | 2.287 |  | 0.003 |  | 13.220 |  |
| RM Factor 1 + group + RM Factor 1 ✻ group |  | 0.200 |  | 0.264 |  | 1.438 |  | 0.004 |  | 2.364 |  |
| RM Factor 1 |  | 0.200 |  | 8.956×10^-4^ |  | 0.004 |  | 1.118 |  | 1.073 |  |
|  | | | | | | | | | | | |

| **Mechanical errors** | | | | | | | | | | | |
| --- | --- | --- | --- | --- | --- | --- | --- | --- | --- | --- | --- |
| **Models** | | **P(M)** | | **P(M\|data)** | | **BF_M_** | | **BF_01_** | | **error %** | |
| Null model (incl. subject and random slopes) |  | 0.200 |  | 0.004 |  | 0.017 |  | 1.000 |  |  |  |
| group |  | 0.200 |  | 0.739 |  | 11.323 |  | 0.006 |  | 0.819 |  |
| RM Factor 1 + group |  | 0.200 |  | 0.189 |  | 0.934 |  | 0.023 |  | 4.135 |  |
| RM Factor 1 + group + RM Factor 1 ✻ group |  | 0.200 |  | 0.066 |  | 0.284 |  | 0.064 |  | 2.024 |  |
| RM Factor 1 |  | 0.200 |  | 0.001 |  | 0.004 |  | 3.961 |  | 1.998 |  |
|  | | | | | | | | | | | |

| **Grammatical errors** | | | | | | | | | | | |
| --- | --- | --- | --- | --- | --- | --- | --- | --- | --- | --- | --- |
| **Models** | | **P(M)** | | **P(M\|data)** | | **BF_M_** | | **BF_01_** | | **error %** | |
| Null model (incl. subject and random slopes) |  | 0.200 |  | 0.008 |  | 0.031 |  | 1.000 |  |  |  |
| group |  | 0.200 |  | 0.692 |  | 8.978 |  | 0.011 |  | 0.888 |  |
| RM Factor 1 + group |  | 0.200 |  | 0.203 |  | 1.018 |  | 0.037 |  | 1.883 |  |
| RM Factor 1 + group + RM Factor 1 ✻ group |  | 0.200 |  | 0.096 |  | 0.423 |  | 0.079 |  | 3.804 |  |
| RM Factor 1 |  | 0.200 |  | 0.002 |  | 0.009 |  | 3.540 |  | 1.018 |  |
|  | | | | | | | | | | | |

| **Text-quality (reader-based)** | | | | | | | | | | | |
| --- | --- | --- | --- | --- | --- | --- | --- | --- | --- | --- | --- |
| **Models** | | **P(M)** | | **P(M\|data)** | | **BF_M_** | | **BF_01_** | | **error %** | |
| Null model (incl. subject and random slopes) |  | 0.200 |  | 0.069 |  | 0.295 |  | 1.000 |  |  |  |
| group |  | 0.200 |  | 0.617 |  | 6.433 |  | 0.111 |  | 2.099 |  |
| RM Factor 1 + group |  | 0.200 |  | 0.173 |  | 0.839 |  | 0.395 |  | 1.655 |  |
| RM Factor 1 + group + RM Factor 1 ✻ group |  | 0.200 |  | 0.122 |  | 0.554 |  | 0.564 |  | 2.060 |  |
| RM Factor 1 |  | 0.200 |  | 0.020 |  | 0.081 |  | 3.471 |  | 1.390 |  |
|  | | | | | | | | | | | |

| **Content** | | | | | | | | | | | |
| --- | --- | --- | --- | --- | --- | --- | --- | --- | --- | --- | --- |
| **Models** | | **P(M)** | | **P(M\|data)** | | **BF_M_** | | **BF_01_** | | **error %** | |
| Null model (incl. subject and random slopes) |  | 0.200 |  | 0.170 |  | 0.820 |  | 1.000 |  |  |  |
| group |  | 0.200 |  | 0.551 |  | 4.905 |  | 0.309 |  | 8.596 |  |
| RM Factor 1 + group |  | 0.200 |  | 0.139 |  | 0.648 |  | 1.220 |  | 2.259 |  |
| RM Factor 1 + group + RM Factor 1 ✻ group |  | 0.200 |  | 0.092 |  | 0.406 |  | 1.847 |  | 1.926 |  |
| RM Factor 1 |  | 0.200 |  | 0.048 |  | 0.200 |  | 3.570 |  | 4.383 |  |
|  | | | | | | | | | | | |

| **Coherence** | | | | | | | | | | | |
| --- | --- | --- | --- | --- | --- | --- | --- | --- | --- | --- | --- |
| **Models** | | **P(M)** | | **P(M\|data)** | | **BF_M_** | | **BF_01_** | | **error %** | |
| Null model (incl. subject and random slopes) |  | 0.200 |  | 0.256 |  | 1.374 |  | 1.000 |  |  |  |
| group |  | 0.200 |  | 0.472 |  | 3.575 |  | 0.542 |  | 0.802 |  |
| RM Factor 1 + group |  | 0.200 |  | 0.121 |  | 0.553 |  | 2.106 |  | 1.477 |  |
| RM Factor 1 + group + RM Factor 1 ✻ group |  | 0.200 |  | 0.086 |  | 0.375 |  | 2.981 |  | 3.099 |  |
| RM Factor 1 |  | 0.200 |  | 0.065 |  | 0.279 |  | 3.922 |  | 2.223 |  |
|  | | | | | | | | | | | |

| **Syntax** | | | | | | | | | | | |
| --- | --- | --- | --- | --- | --- | --- | --- | --- | --- | --- | --- |
| **Models** | | **P(M)** | | **P(M\|data)** | | **BF_M_** | | **BF_01_** | | **error %** | |
| Null model (incl. subject and random slopes) |  | 0.200 |  | 0.048 |  | 0.201 |  | 1.000 |  |  |  |
| group |  | 0.200 |  | 0.682 |  | 8.570 |  | 0.070 |  | 0.953 |  |
| RM Factor 1 + group |  | 0.200 |  | 0.197 |  | 0.982 |  | 0.243 |  | 9.588 |  |
| RM Factor 1 + group + RM Factor 1 ✻ group |  | 0.200 |  | 0.061 |  | 0.259 |  | 0.788 |  | 2.019 |  |
| RM Factor 1 |  | 0.200 |  | 0.013 |  | 0.051 |  | 3.819 |  | 1.026 |  |
|  | | | | | | | | | | | |

| **Vocabulary** | | | | | | | | | | | |
| --- | --- | --- | --- | --- | --- | --- | --- | --- | --- | --- | --- |
| **Models** | | **P(M)** | | **P(M\|data)** | | **BF_M_** | | **BF_01_** | | **error %** | |
| Null model (incl. subject and random slopes) |  | 0.200 |  | 0.008 |  | 0.034 |  | 1.000 |  |  |  |
| group |  | 0.200 |  | 0.611 |  | 6.276 |  | 0.014 |  | 1.734 |  |
| RM Factor 1 + group |  | 0.200 |  | 0.214 |  | 1.090 |  | 0.039 |  | 2.920 |  |
| RM Factor 1 + group + RM Factor 1 ✻ group |  | 0.200 |  | 0.164 |  | 0.784 |  | 0.051 |  | 2.566 |  |
| RM Factor 1 |  | 0.200 |  | 0.003 |  | 0.012 |  | 2.853 |  | 1.628 |  |
|  | | | | | | | | | | | |

*Supplementary Table 3: Text quality: Bayesian mixed ANOVA - Analysis of Effects*

| **Analysis of Effects** | | | | | | | | | | |  |
| --- | --- | --- | --- | --- | --- | --- | --- | --- | --- | --- | --- |
| **Productivity (n of words)** | | | | | | | | | | |  |
| **Effects** | **P(incl)** | | **P(excl)** | | **P(incl\|data)** | | **P(excl\|data)** | | **BF_excl_** | |  |
| RM Factor 1 |  | 0.600 |  | 0.400 |  | 0.385 |  | 0.615 |  | 2.391 |  |
| group |  | 0.600 |  | 0.400 |  | 0.758 |  | 0.242 |  | 0.478 |  |
| RM Factor 1 ✻ group |  | 0.200 |  | 0.800 |  | 0.072 |  | 0.928 |  | 3.214 |  |
|  | | | | | | | | | | |  |
| **Productivity (n of sentences)** | | | | | | | | | | |  |
| **Effects** | **P(incl)** | | **P(excl)** | | **P(incl\|data)** | | **P(excl\|data)** | | **BF_excl_** | |  |
| RM Factor 1 |  | 0.600 |  | 0.400 |  | 0.198 |  | 0.802 |  | 6.080 |  |
| group |  | 0.600 |  | 0.400 |  | 0.668 |  | 0.332 |  | 0.746 |  |
| RM Factor 1 ✻ group |  | 0.200 |  | 0.800 |  | 0.031 |  | 0.969 |  | 7.712 |  |
|  | | | | | | | | | | |  |
| **Lexical complexity (mean word length)** | | | | | | | | | | |  |
| **Effects** | **P(incl)** | | **P(excl)** | | **P(incl\|data)** | | **P(excl\|data)** | | **BF_excl_** | |  |
| RM Factor 1 |  | 0.600 |  | 0.400 |  | 0.999 |  | 0.001 |  | 0.002 |  |
| group |  | 0.600 |  | 0.400 |  | 0.540 |  | 0.460 |  | 1.279 |  |
| RM Factor 1 ✻ group |  | 0.200 |  | 0.800 |  | 0.234 |  | 0.766 |  | 0.820 |  |
|  | | | | | | | | | | |  |
| **Syntactic complexity (average sentence length)** | | | | | | | | | | |  |
| **Effects** | **P(incl)** | | **P(excl)** | | **P(incl\|data)** | | **P(excl\|data)** | | **BF_excl_** | |  |
| RM Factor 1 |  | 0.600 |  | 0.400 |  | 0.247 |  | 0.753 |  | 4.570 |  |
| group |  | 0.600 |  | 0.400 |  | 0.363 |  | 0.637 |  | 2.633 |  |
| RM Factor 1 ✻ group |  | 0.200 |  | 0.800 |  | 0.025 |  | 0.975 |  | 9.913 |  |
|  | | | | | | | | | | |  |
| **Syntactic complexity (n of simple sentences)** | | | | | | | | | | |  |
| **Effects** | **P(incl)** | | **P(excl)** | | **P(incl\|data)** | | **P(excl\|data)** | | **BF_excl_** | |  |
| RM Factor 1 |  | 0.600 |  | 0.400 |  | 0.326 |  | 0.674 |  | 3.103 |  |
| group |  | 0.600 |  | 0.400 |  | 0.488 |  | 0.512 |  | 1.576 |  |
| RM Factor 1 ✻ group |  | 0.200 |  | 0.800 |  | 0.044 |  | 0.956 |  | 5.424 |  |
|  | | | | | | | | | | |  |
| **Syntactic complexity (proportion of simple sentences)** | | | | | | | | | | |  |
| **Effects** | **P(incl)** | | **P(excl)** | | **P(incl\|data)** | | **P(excl\|data)** | | **BF_excl_** | |  |
| RM Factor 1 |  | 0.600 |  | 0.400 |  | 0.275 |  | 0.725 |  | 3.948 |  |
| group |  | 0.600 |  | 0.400 |  | 0.348 |  | 0.652 |  | 2.813 |  |
| RM Factor 1 ✻ group |  | 0.200 |  | 0.800 |  | 0.026 |  | 0.974 |  | 9.225 |  |
|  | | | | | | | | | | |  |
| **Syntactic complexity (n of complex sentences)** | | | | | | | | | | |  |
| **Effects** | **P(incl)** | | **P(excl)** | | **P(incl\|data)** | | **P(excl\|data)** | | **BF_excl_** | |  |
| RM Factor 1 |  | 0.600 |  | 0.400 |  | 0.345 |  | 0.655 |  | 2.852 |  |
| group |  | 0.600 |  | 0.400 |  | 0.668 |  | 0.332 |  | 0.745 |  |
| RM Factor 1 ✻ group |  | 0.200 |  | 0.800 |  | 0.055 |  | 0.945 |  | 4.299 |  |
|  | | | | | | | | | | |  |
| **Syntactic complexity (proportion of complex sentences)** | | | | | | | | | | |  |
| **Effects** | **P(incl)** | | **P(excl)** | | **P(incl\|data)** | | **P(excl\|data)** | | **BF_excl_** | |  |
| RM Factor 1 |  | 0.600 |  | 0.400 |  | 0.271 |  | 0.729 |  | 4.033 |  |
| group |  | 0.600 |  | 0.400 |  | 0.351 |  | 0.649 |  | 2.778 |  |
| RM Factor 1 ✻ group |  | 0.200 |  | 0.800 |  | 0.026 |  | 0.974 |  | 9.397 |  |
|  | | | | | | | | | | |  |
| **Accuracy (n of errors)** | | | | | | | | | | |  |
| **Effects** | **P(incl)** | | **P(excl)** | | **P(incl\|data)** | | **P(excl\|data)** | | **BF_excl_** | |  |
| RM Factor 1 |  | 0.600 |  | 0.400 |  | 0.629 |  | 0.371 |  | 0.884 |  |
| group |  | 0.600 |  | 0.400 |  | 0.998 |  | 0.002 |  | 0.003 |  |
| RM Factor 1 ✻ group |  | 0.200 |  | 0.800 |  | 0.264 |  | 0.736 |  | 0.696 |  |
|  | | | | | | | | | | |  |
| Mechanical errors | | | | | | | | | | |  |
| **Effects** | **P(incl)** | | **P(excl)** | | **P(incl\|data)** | | **P(excl\|data)** | | **BF_excl_** | |  |
| RM Factor 1 |  | 0.600 |  | 0.400 |  | 0.257 |  | 0.743 |  | 4.342 |  |
| group |  | 0.600 |  | 0.400 |  | 0.995 |  | 0.005 |  | 0.008 |  |
| RM Factor 1 ✻ group |  | 0.200 |  | 0.800 |  | 0.066 |  | 0.934 |  | 3.515 |  |
|  | | | | | | | | | | |  |
| **Grammatical errors** | | | | | | | | | | |  |
| **Effects** | **P(incl)** | | **P(excl)** | | **P(incl\|data)** | | **P(excl\|data)** | | **BF_excl_** | |  |
| RM Factor 1 |  | 0.600 |  | 0.400 |  | 0.301 |  | 0.699 |  | 3.490 |  |
| group |  | 0.600 |  | 0.400 |  | 0.990 |  | 0.010 |  | 0.015 |  |
| RM Factor 1 ✻ group |  | 0.200 |  | 0.800 |  | 0.096 |  | 0.904 |  | 2.365 |  |
|  | | | | | | | | | | |  |
| **Text-quality (reader-based)** | | | | | | | | | | |  |
| **Effects** | **P(incl)** | | **P(excl)** | | **P(incl\|data)** | | **P(excl\|data)** | | **BF_excl_** | |  |
| RM Factor 1 |  | 0.600 |  | 0.400 |  | 0.315 |  | 0.685 |  | 3.265 |  |
| group |  | 0.600 |  | 0.400 |  | 0.912 |  | 0.088 |  | 0.145 |  |
| RM Factor 1 ✻ group |  | 0.200 |  | 0.800 |  | 0.122 |  | 0.878 |  | 1.806 |  |
|  | | | | | | | | | | |  |
| **Content** | | | | | | | | | | |  |
| **Effects** | **P(incl)** | | **P(excl)** | | **P(incl\|data)** | | **P(excl\|data)** | | **BF_excl_** | |  |
| RM Factor 1 |  | 0.600 |  | 0.400 |  | 0.279 |  | 0.721 |  | 3.874 |  |
| group |  | 0.600 |  | 0.400 |  | 0.782 |  | 0.218 |  | 0.417 |  |
| RM Factor 1 ✻ group |  | 0.200 |  | 0.800 |  | 0.092 |  | 0.908 |  | 2.465 |  |
|  | | | | | | | | | | |  |
| **Coherence** | | | | | | | | | | |  |
| **Effects** | **P(incl)** | | **P(excl)** | | **P(incl\|data)** | | **P(excl\|data)** | | **BF_excl_** | |  |
| RM Factor 1 |  | 0.600 |  | 0.400 |  | 0.272 |  | 0.728 |  | 4.007 |  |
| group |  | 0.600 |  | 0.400 |  | 0.679 |  | 0.321 |  | 0.709 |  |
| RM Factor 1 ✻ group |  | 0.200 |  | 0.800 |  | 0.086 |  | 0.914 |  | 2.665 |  |
|  | | | | | | | | | | |  |
| **Syntax** | | | | | | | | | | |  |
| **Effects** | **P(incl)** | | **P(excl)** | | **P(incl\|data)** | | **P(excl\|data)** | | **BF_excl_** | |  |
| RM Factor 1 |  | 0.600 |  | 0.400 |  | 0.270 |  | 0.730 |  | 4.048 |  |
| group |  | 0.600 |  | 0.400 |  | 0.940 |  | 0.060 |  | 0.096 |  |
| RM Factor 1 ✻ group |  | 0.200 |  | 0.800 |  | 0.061 |  | 0.939 |  | 3.864 |  |
|  | | | | | | | | | | |  |
| **Vocabulary** | | | | | | | | | | |  |
| **Effects** | **P(incl)** | | **P(excl)** | | **P(incl\|data)** | | **P(excl\|data)** | | **BF_excl_** | |  |
| RM Factor 1 |  | 0.600 |  | 0.400 |  | 0.381 |  | 0.619 |  | 2.438 |  |
| group |  | 0.600 |  | 0.400 |  | 0.989 |  | 0.011 |  | 0.017 |  |
| RM Factor 1 ✻ group |  | 0.200 |  | 0.800 |  | 0.164 |  | 0.836 |  | 1.276 |  |
|  | | | | | | | | | | |  |

*Supplementary Table 4: Writing process: Bayesian mixed ANOVA - Model Comparison*

| **Total process time (s)** | | | | | | | | | | | |
| --- | --- | --- | --- | --- | --- | --- | --- | --- | --- | --- | --- |
| **Models** | | **P(M)** | | **P(M\|data)** | | **BF_M_** | | **BF_01_** | | **error %** | |
| Null model (incl. subject and random slopes) |  | 0.200 |  | 0.507 |  | 4.118 |  | 1.000 |  |  |  |
| group |  | 0.200 |  | 0.251 |  | 1.339 |  | 2.023 |  | 0.690 |  |
| RM Factor 1 |  | 0.200 |  | 0.146 |  | 0.684 |  | 3.472 |  | 1.352 |  |
| RM Factor 1 + group |  | 0.200 |  | 0.072 |  | 0.312 |  | 7.010 |  | 1.371 |  |
| RM Factor 1 + group + RM Factor 1 ✻ group |  | 0.200 |  | 0.023 |  | 0.096 |  | 21.598 |  | 2.026 |  |
|  | | | | | | | | | | | |

| **Total Active writing time (s)** | | | | | | | | | | | |
| --- | --- | --- | --- | --- | --- | --- | --- | --- | --- | --- | --- |
| **Models** | | **P(M)** | | **P(M\|data)** | | **BF_M_** | | **BF_01_** | | **error %** | |
| Null model (incl. subject and random slopes) |  | 0.200 |  | 0.163 |  | 0.780 |  | 1.000 |  |  |  |
| group |  | 0.200 |  | 0.564 |  | 5.179 |  | 0.289 |  | 1.538 |  |
| RM Factor 1 + group |  | 0.200 |  | 0.136 |  | 0.627 |  | 1.205 |  | 1.978 |  |
| RM Factor 1 + group + RM Factor 1 ✻ group |  | 0.200 |  | 0.092 |  | 0.407 |  | 1.769 |  | 3.146 |  |
| RM Factor 1 |  | 0.200 |  | 0.045 |  | 0.187 |  | 3.650 |  | 4.462 |  |
|  | | | | | | | | | | | |

| **Revisions - Insertions** | | | | | | | | | | | |
| --- | --- | --- | --- | --- | --- | --- | --- | --- | --- | --- | --- |
| **Models** | | **P(M)** | | **P(M\|data)** | | **BF_M_** | | **BF_01_** | | **error %** | |
| Null model (incl. subject and random slopes) |  | 0.200 |  | 2.760×10^-4^ |  | 0.001 |  | 1.000 |  |  |  |
| RM Factor 1 |  | 0.200 |  | 0.681 |  | 8.555 |  | 4.051×10^-4^ |  | 2.073 |  |
| RM Factor 1 + group |  | 0.200 |  | 0.236 |  | 1.233 |  | 0.001 |  | 2.763 |  |
| RM Factor 1 + group + RM Factor 1 ✻ group |  | 0.200 |  | 0.083 |  | 0.360 |  | 0.003 |  | 2.745 |  |
| group |  | 0.200 |  | 9.539×10^-5^ |  | 3.816×10^-4^ |  | 2.894 |  | 3.567 |  |
|  | | | | | | | | | | | |

| **Revisions - Deletions** | | | | | | | | | | | |
| --- | --- | --- | --- | --- | --- | --- | --- | --- | --- | --- | --- |
| **Models** | | **P(M)** | | **P(M\|data)** | | **BF_M_** | | **BF_01_** | | **error %** | |
| Null model (incl. subject and random slopes) |  | 0.200 |  | 3.562×10^-6^ |  | 1.425×10^-5^ |  | 1.000 |  |  |  |
| RM Factor 1 |  | 0.200 |  | 0.565 |  | 5.202 |  | 6.301×10^-6^ |  | 3.631 |  |
| RM Factor 1 + group |  | 0.200 |  | 0.324 |  | 1.919 |  | 1.099×10^-5^ |  | 4.531 |  |
| RM Factor 1 + group + RM Factor 1 ✻ group |  | 0.200 |  | 0.110 |  | 0.497 |  | 3.225×10^-5^ |  | 1.602 |  |
| group |  | 0.200 |  | 1.654×10^-6^ |  | 6.616×10^-6^ |  | 2.154 |  | 1.781 |  |
|  | | | | | | | | | | | |

| **Number of R-bursts** | | | | | | | | | | | |
| --- | --- | --- | --- | --- | --- | --- | --- | --- | --- | --- | --- |
| **Models** | | **P(M)** | | **P(M\|data)** | | **BF_M_** | | **BF_01_** | | **error %** | |
| Null model (incl. subject and random slopes) |  | 0.200 |  | 0.297 |  | 1.686 |  | 1.000 |  |  |  |
| RM Factor 1 |  | 0.200 |  | 0.369 |  | 2.334 |  | 0.805 |  | 1.468 |  |
| RM Factor 1 + group |  | 0.200 |  | 0.155 |  | 0.733 |  | 1.915 |  | 2.749 |  |
| group |  | 0.200 |  | 0.124 |  | 0.565 |  | 2.396 |  | 0.964 |  |
| RM Factor 1 + group + RM Factor 1 ✻ group |  | 0.200 |  | 0.056 |  | 0.239 |  | 5.268 |  | 3.039 |  |
|  | | | | | | | | | | | |

| **Mean R-bursts time (s)** | | | | | | | | | | | |
| --- | --- | --- | --- | --- | --- | --- | --- | --- | --- | --- | --- |
| **Models** | | **P(M)** | | **P(M\|data)** | | **BF_M_** | | **BF_01_** | | **error %** | |
| Null model (incl. subject and random slopes) |  | 0.200 |  | 0.004 |  | 0.014 |  | 1.000 |  |  |  |
| RM Factor 1 |  | 0.200 |  | 0.585 |  | 5.640 |  | 0.006 |  | 1.058 |  |
| RM Factor 1 + grupa |  | 0.200 |  | 0.303 |  | 1.740 |  | 0.012 |  | 1.881 |  |
| RM Factor 1 + grupa + RM Factor 1 ✻  grupa |  | 0.200 |  | 0.107 |  | 0.478 |  | 0.033 |  | 2.296 |  |
| grupa |  | 0.200 |  | 0.002 |  | 0.006 |  | 2.266 |  | 0.660 |  |
|  | | | | | | | | | | | |
| *Note.*  All models include subject, and random slopes for all repeated measures factors. | | | | | | | | | | | |

| **Total number of characters (incl. spaces)** | | | | | | | | | | | |
| --- | --- | --- | --- | --- | --- | --- | --- | --- | --- | --- | --- |
| **Models** | | **P(M)** | | **P(M\|data)** | | **BF_M_** | | **BF_01_** | | **error %** | |
| Null model (incl. subject and random slopes) |  | 0.200 |  | 0.109 |  | 0.487 |  | 1.000 |  |  |  |
| group |  | 0.200 |  | 0.386 |  | 2.515 |  | 0.281 |  | 2.399 |  |
| RM Factor 1 + group |  | 0.200 |  | 0.311 |  | 1.803 |  | 0.349 |  | 2.414 |  |
| RM Factor 1 + group + RM Factor 1 ✻ group |  | 0.200 |  | 0.116 |  | 0.527 |  | 0.933 |  | 7.122 |  |
| RM Factor 1 |  | 0.200 |  | 0.078 |  | 0.340 |  | 1.386 |  | 0.755 |  |
|  | | | | | | | | | | | |

| **Number of characters per min. (incl. spaces)** | | | | | | | | | | | |
| --- | --- | --- | --- | --- | --- | --- | --- | --- | --- | --- | --- |
| **Models** | | **P(M)** | | **P(M\|data)** | | **BF_M_** | | **BF_01_** | | **error %** | |
| Null model (incl. subject and random slopes) |  | 0.200 |  | 0.008 |  | 0.030 |  | 1.000 |  |  |  |
| RM Factor 1 + group |  | 0.200 |  | 0.445 |  | 3.212 |  | 0.017 |  | 4.250 |  |
| RM Factor 1 + group + RM Factor 1 ✻ group |  | 0.200 |  | 0.329 |  | 1.960 |  | 0.023 |  | 1.724 |  |
| group |  | 0.200 |  | 0.201 |  | 1.007 |  | 0.037 |  | 1.282 |  |
| RM Factor 1 |  | 0.200 |  | 0.017 |  | 0.070 |  | 0.438 |  | 0.899 |  |
|  | | | | | | | | | | | |

| **Total number of characters (excl. spaces)** | | | | | | | | | | | |
| --- | --- | --- | --- | --- | --- | --- | --- | --- | --- | --- | --- |
| **Models** | | **P(M)** | | **P(M\|data)** | | **BF_M_** | | **BF_01_** | | **error %** | |
| Null model (incl. subject and random slopes) |  | 0.200 |  | 0.082 |  | 0.357 |  | 1.000 |  |  |  |
| group |  | 0.200 |  | 0.386 |  | 2.518 |  | 0.212 |  | 2.223 |  |
| RM Factor 1 + group |  | 0.200 |  | 0.313 |  | 1.823 |  | 0.262 |  | 2.802 |  |
| RM Factor 1 + group + RM Factor 1 ✻ group |  | 0.200 |  | 0.152 |  | 0.718 |  | 0.538 |  | 13.335 |  |
| RM Factor 1 |  | 0.200 |  | 0.067 |  | 0.285 |  | 1.230 |  | 0.712 |  |
|  | | | | | | | | | | | |

| **Number of characters per min. (excl. spaces)** | | | | | | | | | | | |
| --- | --- | --- | --- | --- | --- | --- | --- | --- | --- | --- | --- |
| **Models** | | **P(M)** | | **P(M\|data)** | | **BF_M_** | | **BF_01_** | | **error %** | |
| Null model (incl. subject and random slopes) |  | 0.200 |  | 0.006 |  | 0.025 |  | 1.000 |  |  |  |
| RM Factor 1 + group |  | 0.200 |  | 0.414 |  | 2.822 |  | 0.015 |  | 1.720 |  |
| RM Factor 1 + group + RM Factor 1 ✻ group |  | 0.200 |  | 0.375 |  | 2.404 |  | 0.016 |  | 3.751 |  |
| group |  | 0.200 |  | 0.190 |  | 0.941 |  | 0.032 |  | 1.241 |  |
| RM Factor 1 |  | 0.200 |  | 0.014 |  | 0.058 |  | 0.432 |  | 0.827 |  |
|  | | | | | | | | | | | |

| **Total keystrokes** | | | | | | | | | | | |
| --- | --- | --- | --- | --- | --- | --- | --- | --- | --- | --- | --- |
| **Models** | | **P(M)** | | **P(M\|data)** | | **BF_M_** | | **BF_01_** | | **error %** | |
| Null model (incl. subject and random slopes) |  | 0.200 |  | 0.177 |  | 0.862 |  | 1.000 |  |  |  |
| group |  | 0.200 |  | 0.543 |  | 4.744 |  | 0.327 |  | 1.144 |  |
| RM Factor 1 + group |  | 0.200 |  | 0.169 |  | 0.815 |  | 1.048 |  | 1.669 |  |
| RM Factor 1 + group + RM Factor 1 ✻ group |  | 0.200 |  | 0.056 |  | 0.236 |  | 3.179 |  | 2.462 |  |
| RM Factor 1 |  | 0.200 |  | 0.055 |  | 0.233 |  | 3.216 |  | 0.980 |  |
|  | | | | | | | | | | | |

| **Total non-character keys** | | | | | | | | | | | |
| --- | --- | --- | --- | --- | --- | --- | --- | --- | --- | --- | --- |
| **Models** | | **P(M)** | | **P(M\|data)** | | **BF_M_** | | **BF_01_** | | **error %** | |
| Null model (incl. subject and random slopes) |  | 0.200 |  | 4.525×10^-16^ |  | 1.810×10^-15^ |  | 1.000 |  |  |  |
| RM Factor 1 + group |  | 0.200 |  | 0.529 |  | 4.488 |  | 8.558×10^-16^ |  | 2.780 |  |
| RM Factor 1 |  | 0.200 |  | 0.282 |  | 1.572 |  | 1.604×10^-15^ |  | 1.169 |  |
| RM Factor 1 + group + RM Factor 1 ✻ group |  | 0.200 |  | 0.189 |  | 0.933 |  | 2.392×10^-15^ |  | 5.368 |  |
| group |  | 0.200 |  | 4.302×10^-16^ |  | 1.721×10^-15^ |  | 1.052 |  | 3.298 |  |
|  | | | | | | | | | | | |

| **Total typed (incl. spaces)** | | | | | | | | | | | |
| --- | --- | --- | --- | --- | --- | --- | --- | --- | --- | --- | --- |
| **Models** | | **P(M)** | | **P(M\|data)** | | **BF_M_** | | **BF_01_** | | **error %** | |
| Null model (incl. subject and random slopes) |  | 0.200 |  | 0.119 |  | 0.541 |  | 1.000 |  |  |  |
| group |  | 0.200 |  | 0.494 |  | 3.905 |  | 0.241 |  | 2.623 |  |
| RM Factor 1 + group |  | 0.200 |  | 0.229 |  | 1.191 |  | 0.519 |  | 3.461 |  |
| RM Factor 1 + group + RM Factor 1 ✻ group |  | 0.200 |  | 0.100 |  | 0.442 |  | 1.197 |  | 8.877 |  |
| RM Factor 1 |  | 0.200 |  | 0.058 |  | 0.246 |  | 2.060 |  | 1.192 |  |
|  | | | | | | | | | | | |

| **Total typed per min. (incl. spaces)** | | | | | | | | | | | |
| --- | --- | --- | --- | --- | --- | --- | --- | --- | --- | --- | --- |
| **Models** | | **P(M)** | | **P(M\|data)** | | **BF_M_** | | **BF_01_** | | **error %** | |
| Null model (incl. subject and random slopes) |  | 0.200 |  | 0.004 |  | 0.014 |  | 1.000 |  |  |  |
| RM Factor 1 + group + RM Factor 1 ✻ group |  | 0.200 |  | 0.474 |  | 3.603 |  | 0.007 |  | 2.086 |  |
| RM Factor 1 + group |  | 0.200 |  | 0.372 |  | 2.368 |  | 0.009 |  | 1.420 |  |
| group |  | 0.200 |  | 0.141 |  | 0.656 |  | 0.025 |  | 1.294 |  |
| RM Factor 1 |  | 0.200 |  | 0.010 |  | 0.040 |  | 0.355 |  | 0.868 |  |
|  | | | | | | | | | | | |

| **Total typed (excl. spaces)** | | | | | | | | | | | |
| --- | --- | --- | --- | --- | --- | --- | --- | --- | --- | --- | --- |
| **Models** | | **P(M)** | | **P(M\|data)** | | **BF_M_** | | **BF_01_** | | **error %** | |
| Null model (incl. subject and random slopes) |  | 0.200 |  | 0.079 |  | 0.341 |  | 1.000 |  |  |  |
| group |  | 0.200 |  | 0.379 |  | 2.439 |  | 0.208 |  | 2.007 |  |
| RM Factor 1 + group |  | 0.200 |  | 0.342 |  | 2.078 |  | 0.230 |  | 3.510 |  |
| RM Factor 1 + group + RM Factor 1 ✻ group |  | 0.200 |  | 0.131 |  | 0.604 |  | 0.600 |  | 3.412 |  |
| RM Factor 1 |  | 0.200 |  | 0.070 |  | 0.299 |  | 1.130 |  | 1.053 |  |
|  | | | | | | | | | | | |

| **Total typed per min. (excl. spaces)** | | | | | | | | | | | |
| --- | --- | --- | --- | --- | --- | --- | --- | --- | --- | --- | --- |
| **Models** | | **P(M)** | | **P(M\|data)** | | **BF_M_** | | **BF_01_** | | **error %** | |
| Null model (incl. subject and random slopes) |  | 0.200 |  | 9.994×10^-4^ |  | 0.004 |  | 1.000 |  |  |  |
| RM Factor 1 + group + RM Factor 1 ✻ group |  | 0.200 |  | 0.537 |  | 4.647 |  | 0.002 |  | 2.240 |  |
| RM Factor 1 + group |  | 0.200 |  | 0.404 |  | 2.716 |  | 0.002 |  | 1.697 |  |
| group |  | 0.200 |  | 0.048 |  | 0.202 |  | 0.021 |  | 0.991 |  |
| RM Factor 1 |  | 0.200 |  | 0.009 |  | 0.037 |  | 0.108 |  | 2.691 |  |
|  | | | | | | | | | | | |

| **Produced ratio (incl. spaces)** | | | | | | | | | | | |
| --- | --- | --- | --- | --- | --- | --- | --- | --- | --- | --- | --- |
| **Models** | | **P(M)** | | **P(M\|data)** | | **BF_M_** | | **BF_01_** | | **error %** | |
| Null model (incl. subject and random slopes) |  | 0.200 |  | 0.371 |  | 2.360 |  | 1.000 |  |  |  |
| group |  | 0.200 |  | 0.273 |  | 1.503 |  | 1.359 |  | 1.766 |  |
| RM Factor 1 |  | 0.200 |  | 0.175 |  | 0.847 |  | 2.123 |  | 0.732 |  |
| RM Factor 1 + group |  | 0.200 |  | 0.131 |  | 0.601 |  | 2.840 |  | 1.382 |  |
| RM Factor 1 + group + RM Factor 1 ✻ group |  | 0.200 |  | 0.050 |  | 0.213 |  | 7.355 |  | 5.854 |  |
|  | | | | | | | | | | | |

| **Characters (incl. spaces)** | | | | | | | | | | | |
| --- | --- | --- | --- | --- | --- | --- | --- | --- | --- | --- | --- |
| **Models** | | **P(M)** | | **P(M\|data)** | | **BF_M_** | | **BF_01_** | | **error %** | |
| Null model (incl. subject and random slopes) |  | 0.200 |  | 0.349 |  | 2.148 |  | 1.000 |  |  |  |
| group |  | 0.200 |  | 0.295 |  | 1.675 |  | 1.183 |  | 2.083 |  |
| RM Factor 1 |  | 0.200 |  | 0.170 |  | 0.819 |  | 2.056 |  | 4.120 |  |
| RM Factor 1 + group |  | 0.200 |  | 0.136 |  | 0.632 |  | 2.559 |  | 3.647 |  |
| RM Factor 1 + group + RM Factor 1 ✻ group |  | 0.200 |  | 0.049 |  | 0.206 |  | 7.120 |  | 11.617 |  |
|  | | | | | | | | | | | |

| **Characters (excl. spaces)** | | | | | | | | | | | |
| --- | --- | --- | --- | --- | --- | --- | --- | --- | --- | --- | --- |
| **Models** | | **P(M)** | | **P(M\|data)** | | **BF_M_** | | **BF_01_** | | **error %** | |
| Null model (incl. subject and random slopes) |  | 0.200 |  | 0.437 |  | 3.109 |  | 1.000 |  |  |  |
| group |  | 0.200 |  | 0.340 |  | 2.058 |  | 1.287 |  | 2.009 |  |
| RM Factor 1 |  | 0.200 |  | 0.106 |  | 0.472 |  | 4.144 |  | 1.440 |  |
| RM Factor 1 + group |  | 0.200 |  | 0.092 |  | 0.405 |  | 4.758 |  | 10.726 |  |
| RM Factor 1 + group + RM Factor 1 ✻ group |  | 0.200 |  | 0.025 |  | 0.104 |  | 17.181 |  | 2.287 |  |
|  | | | | | | | | | | | |

| **Words** | | | | | | | | | | | |
| --- | --- | --- | --- | --- | --- | --- | --- | --- | --- | --- | --- |
| **Models** | | **P(M)** | | **P(M\|data)** | | **BF_M_** | | **BF_01_** | | **error %** | |
| Null model (incl. subject and random slopes) |  | 0.200 |  | 8.452×10^-5^ |  | 3.381×10^-4^ |  | 1.000 |  |  |  |
| RM Factor 1 + group |  | 0.200 |  | 0.514 |  | 4.228 |  | 1.645×10^-4^ |  | 1.716 |  |
| RM Factor 1 |  | 0.200 |  | 0.322 |  | 1.902 |  | 2.623×10^-4^ |  | 1.415 |  |
| RM Factor 1 + group + RM Factor 1 ✻ group |  | 0.200 |  | 0.164 |  | 0.783 |  | 5.164×10^-4^ |  | 2.587 |  |
| group |  | 0.200 |  | 1.427×10^-4^ |  | 5.711×10^-4^ |  | 0.592 |  | 1.374 |  |
|  | | | | | | | | | | | |

*Supplementary Table 5: Writing process: Bayesian mixed ANOVA - Analysis of Effects*

| **Total process time (s)** | | | | | | | | | | | | | | | | | | | | |  |  |  |  |  |  |
| --- | --- | --- | --- | --- | --- | --- | --- | --- | --- | --- | --- | --- | --- | --- | --- | --- | --- | --- | --- | --- | --- | --- | --- | --- | --- | --- |
| **Effects** | | **P(incl)** | | | **P(excl)** | | | **P(incl\|data)** | | | | **P(excl\|data)** | | | | **BF_excl_** | | | | |  |  |  |  |  |  |
| RM Factor 1 |  | 0.600 | |  | 0.400 | |  | 0.242 | | |  | 0.758 | | |  | 4.699 | | | | |  |  |  |  |  |  |
| group |  | 0.600 | |  | 0.400 | |  | 0.347 | | |  | 0.653 | | |  | 2.828 | | | | |  |  |  |  |  |  |
| RM Factor 1 ✻ group |  | 0.200 | |  | 0.800 | |  | 0.023 | | |  | 0.977 | | |  | 10.394 | | | | |  |  |  |  |  |  |
|  | | | | | | | | | | | | | | | | | | | | |  |  |  |  |  |  |
| **Total Active writing time (s)** | | | | | | | | | | | | | | | | | | | |  |  |  |  |  |  |  |
| **Effects** | | **P(incl)** | | | **P(excl)** | | | **P(incl\|data)** | | | | **P(excl\|data)** | | | | **BF_excl_** | | | |  |  |  |  |  |  |  |
| RM Factor 1 |  | 0.600 | |  | 0.400 | |  | 0.273 | | |  | 0.727 | | |  | 4.004 | | | |  |  |  |  |  |  |  |
| group |  | 0.600 | |  | 0.400 | |  | 0.792 | | |  | 0.208 | | |  | 0.394 | | | |  |  |  |  |  |  |  |
| RM Factor 1 ✻ group |  | 0.200 | |  | 0.800 | |  | 0.092 | | |  | 0.908 | | |  | 2.459 | | | |  |  |  |  |  |  |  |
|  | | | | | | | | | | | | | | | | | | | |  |  |  |  |  |  |  |
|  | | | | | | | | | | | | | | | | | | | |  |  |  |  |  |  |  |
| **Revisions - Insertions** | | | | | | | | | | | | | | | | | | | | | | |  |  |  |  |
| **Effects** | **P(incl)** | | **P(excl)** | | | **P(incl\|data)** | | | | **P(excl\|data)** | | | | **BF_excl_** | | | | | | | | |  |  |  |  |
| RM Factor 1 |  | 0.600 |  | 0.400 | |  | 1.000 | | |  | 3.714×10^-4^ | | | |  | 5.573×10^-4^ | | | | | | | |  | |  |
| group |  | 0.600 |  | 0.400 | |  | 0.318 | | |  | 0.682 | | | |  | 3.212 | | | | | | | |  | |  |
| RM Factor 1 ✻ group |  | 0.200 |  | 0.800 | |  | 0.083 | | |  | 0.917 | | | |  | 2.776 | | | | | | | |  | |  |
|  | | | | | | | | | | | | | | | | | | | | | | | | |  |  |
| **Revisions - Deletions** | | | | | | | | | | | | | | | | | | | | | | |  |  |  |  |
| **Effects** | **P(incl)** | | **P(excl)** | | | **P(incl\|data)** | | | | **P(excl\|data)** | | | | **BF_excl_** | | | | | | | | |  |  |  |  |
| RM Factor 1 |  | 0.600 |  | 0.400 | |  | 1.000 | | |  | 5.216×10^-6^ | | | |  | 7.824×10^-6^ | | | | | | | |  |  |  |
| group |  | 0.600 |  | 0.400 | |  | 0.435 | | |  | 0.565 | | | |  | 1.951 | | | | | | | |  |  |  |
| RM Factor 1 ✻ group |  | 0.200 |  | 0.800 | |  | 0.110 | | |  | 0.890 | | | |  | 2.013 | | | | | | | |  |  |  |
|  | | | | | | | | | | | | | | | | | | | | | | | |  |  |  |
| **Number of R-bursts** | | | | | | | | | | | | | | | | |  |  |  |  |  |  |  |  |  |  |
| **Effects** | | **P(incl)** | | | **P(excl)** | | | **P(incl\|data)** | | | | **P(excl\|data)** | | | | **BF_excl_** | | | | | | | | |  |  |
| RM Factor 1 |  | 0.600 | |  | 0.400 | | |  | 0.580 | | | |  | 0.420 | | |  | 1.088 | | | |  |  |  |  |  |
| group |  | 0.600 | |  | 0.400 | | |  | 0.335 | | | |  | 0.665 | | |  | 2.979 | | | |  |  |  |  |  |
| RM Factor 1 ✻ group |  | 0.200 | |  | 0.800 | | |  | 0.056 | | | |  | 0.944 | | |  | 4.191 | | | |  |  |  |  |  |
|  | | | | | | | | | | | | | | | | | | | | | | | | |  |  |
| \| **Mean R-bursts time (s)** \| \| \| \| \| \| \| \| \| \| \| \| \| --- \| --- \| --- \| --- \| --- \| --- \| --- \| --- \| --- \| --- \| --- \| --- \| \| **Effects** \| \| **P(incl)** \| \| **P(excl)** \| \| **P(incl\|data)** \| \| **P(excl\|data)** \| \| **BF_excl_** \| \| \| RM Factor 1 \|  \| 0.600 \|  \| 0.400 \|  \| 0.995 \|  \| 0.005 \|  \| 0.008 \|  \| \| grupa \|  \| 0.600 \|  \| 0.400 \|  \| 0.411 \|  \| 0.589 \|  \| 2.146 \|  \| \| RM Factor 1 ✻  grupa \|  \| 0.200 \|  \| 0.800 \|  \| 0.107 \|  \| 0.893 \|  \| 2.093 \|  \| \|  \| \| \| \| \| \| \| \| \| \| \| \| | | | | | | | | | | | | | | | | | | | |  |  |  |  |  |  |  |
| **Total number of characters (incl. spaces)** | | | | | | | | | | | | | | | | | | | |  |  |  |  |  |  |  |
| **Effects** | | **P(incl)** | | | **P(excl)** | | | **P(incl\|data)** | | | | **P(excl\|data)** | | | | **BF_excl_** | | | |  |  |  |  |  |  |  |
| RM Factor 1 |  | 0.600 | |  | 0.400 | |  | 0.505 | | |  | 0.495 | | |  | 1.468 | | | |  |  |  |  |  |  |  |
| group |  | 0.600 | |  | 0.400 | |  | 0.813 | | |  | 0.187 | | |  | 0.345 | | | |  |  |  |  |  |  |  |
| RM Factor 1 ✻ group |  | 0.200 | |  | 0.800 | |  | 0.116 | | |  | 0.884 | | |  | 1.899 | | | |  |  |  |  |  |  |  |
|  | | | | | | | | | | | | | | | | | | | |  |  |  |  |  |  |  |
| **Number of characters per min. (incl. spaces)** | | | | | | | | | | | | | | | | | | | |  |  |  |  |  |  |  |
| **Effects** | | **P(incl)** | | | **P(excl)** | | | **P(incl\|data)** | | | | **P(excl\|data)** | | | | **BF_excl_** | | | |  |  |  |  |  |  |  |
| RM Factor 1 |  | 0.600 | |  | 0.400 | |  | 0.791 | | |  | 0.209 | | |  | 0.395 | | |  |  |  |  |  |  |  |  |
| group |  | 0.600 | |  | 0.400 | |  | 0.975 | | |  | 0.025 | | |  | 0.038 | | |  |  |  |  |  |  |  |  |
| RM Factor 1 ✻ group |  | 0.200 | |  | 0.800 | |  | 0.329 | | |  | 0.671 | | |  | 0.510 | | |  |  |  |  |  |  |  |  |
|  | | | | | | | | | | | | | | | | | | | |  |  |  |  |  |  |  |
| **Total number of characters (excl. spaces)** | | | | | | | | | | | | | | | | | | |  |  |  |  |  |  |  |  |
| **Effects** | | **P(incl)** | | **P(excl)** | | | **P(incl\|data)** | | | | | **P(excl\|data)** | | | **BF_excl_** | | | |  |  |  |  |  |  |  |  |
| RM Factor 1 |  | 0.600 |  | 0.400 | |  | 0.532 | | |  | | 0.468 | |  | 1.321 | | |  |  |  |  |  |  |  |  |  |
| group |  | 0.600 |  | 0.400 | |  | 0.851 | | |  | | 0.149 | |  | 0.262 | | |  |  |  |  |  |  |  |  |  |
| RM Factor 1 ✻ group |  | 0.200 |  | 0.800 | |  | 0.152 | | |  | | 0.848 | |  | 1.393 | | |  |  |  |  |  |  |  |  |  |
|  | | | | | | | | | | | | | | | | | | |  |  |  |  |  |  |  |  |
| **Number of characters per min. (excl. spaces)** | | | | | | | | | | | | | | | | | |  |  |  |  |  |  |  |  |  |
| **Effects** | | **P(incl)** | | **P(excl)** | | | **P(incl\|data)** | | | | **P(excl\|data)** | | | | **BF_excl_** | | |  |  |  |  |  |  |  |  |  |
| RM Factor 1 |  | 0.600 |  | 0.400 | |  | 0.803 | |  | | 0.197 | |  | | 0.367 | |  |  |  |  |  |  |  |  |  |  |
| group |  | 0.600 |  | 0.400 | |  | 0.980 | |  | | 0.020 | |  | | 0.031 | |  |  |  |  |  |  |  |  |  |  |
| RM Factor 1 ✻ group |  | 0.200 |  | 0.800 | |  | 0.375 | |  | | 0.625 | |  | | 0.416 | |  |  |  |  |  |  |  |  |  |  |
|  | | | | | | | | | | | | | | | | | |  |  |  |  |  |  |  |  |  |
| **Total keystrokes** | | | | | | | | | | | | | | | | | |  |  |  |  |  |  |  |  |  |
| **Effects** | | **P(incl)** | | **P(excl)** | | **P(incl\|data)** | | | | **P(excl\|data)** | | | | **BF_excl_** | | | |  |  |  |  |  |  |  |  |  |
| RM Factor 1 |  | 0.600 |  | 0.400 | |  | 0.280 | | |  | 0.720 | | |  | 3.855 | | |  |  |  |  |  |  |  |  |  |
| group |  | 0.600 |  | 0.400 | |  | 0.768 | | |  | 0.232 | | |  | 0.454 | | |  |  |  |  |  |  |  |  |  |
| RM Factor 1 ✻ group |  | 0.200 |  | 0.800 | |  | 0.056 | | |  | 0.944 | | |  | 4.231 | | |  |  |  |  |  |  |  |  |  |
|  | | | | | | | | | | | | | | | | | |  |  |  |  |  |  |  |  |  |
| **Total non-character keys** | | | | | | | | | | | | | | | | |  |  |  |  |  |  |  |  |  |  |
| **Effects** | **P(incl)** | | **P(excl)** | | | **P(incl\|data)** | | | | **P(excl\|data)** | | | | **BF_excl_** | | |  |  |  |  |  |  |  |  |  |  |
| RM Factor 1 |  | 0.600 |  | 0.400 | |  | 1.000 | | |  | 0.000 | | |  | 0.000 | | |  |  |  |  |  |  |  |  |  |
| group |  | 0.600 |  | 0.400 | |  | 0.718 | | |  | 0.282 | | |  | 0.589 | | |  |  |  |  |  |  |  |  |  |
| RM Factor 1 ✻ group |  | 0.200 |  | 0.800 | |  | 0.189 | | |  | 0.811 | | |  | 1.071 | | |  |  |  |  |  |  |  |  |  |
|  | | | | | | | | | | | | | | | | | |  |  |  |  |  |  |  |  |  |
| **Total typed (incl. spaces)** | | | | | | | | | | | | | | | | |  |  |  |  |  |  |  |  |  |  |
| **Effects** | **P(incl)** | | **P(excl)** | | | **P(incl\|data)** | | | | **P(excl\|data)** | | | | **BF_excl_** | | |  |  |  |  |  |  |  |  |  |  |
| RM Factor 1 |  | 0.600 |  | 0.400 | |  | 0.387 | | |  | 0.613 | | |  | 2.377 | | |  |  |  |  |  |  |  |  |  |
| group |  | 0.600 |  | 0.400 | |  | 0.823 | | |  | 0.177 | | |  | 0.323 | | |  |  |  |  |  |  |  |  |  |
| RM Factor 1 ✻ group |  | 0.200 |  | 0.800 | |  | 0.100 | | |  | 0.900 | | |  | 2.262 | | |  |  |  |  |  |  |  |  |  |
|  | | | | | | | | | | | | | | | | | |  |  |  |  |  |  |  |  |  |
| **Total typed per min. (incl. spaces)** | | | | | | | | | | | | | | | | |  |  |  |  |  |  |  |  |  |  |
| **Effects** | **P(incl)** | | **P(excl)** | | | **P(incl\|data)** | | | | **P(excl\|data)** | | | | **BF_excl_** | | |  |  |  |  |  |  |  |  |  |  |
| RM Factor 1 |  | 0.600 |  | 0.400 | |  | 0.856 | | |  | 0.144 | | |  | 0.253 | | |  |  |  |  |  |  |  |  |  |
| group |  | 0.600 |  | 0.400 | |  | 0.987 | | |  | 0.013 | | |  | 0.020 | | |  |  |  |  |  |  |  |  |  |
| RM Factor 1 ✻ group |  | 0.200 |  | 0.800 | |  | 0.474 | | |  | 0.526 | | |  | 0.278 | | |  |  |  |  |  |  |  |  |  |
|  | | | | | | | | | | | | | | | | | |  |  |  |  |  |  |  |  |  |
| **Total typed (excl. spaces)** | | | | | | | | | | | | | | | | |  |  |  |  |  |  |  |  |  |  |
| **Effects** | **P(incl)** | | **P(excl)** | | | **P(incl\|data)** | | | | **P(excl\|data)** | | | | **BF_excl_** | | |  |  |  |  |  |  |  |  |  |  |
| RM Factor 1 |  | 0.600 |  | 0.400 | |  | 0.543 | | |  | 0.457 | | |  | 1.264 | | |  |  |  |  |  |  |  |  |  |
| group |  | 0.600 |  | 0.400 | |  | 0.852 | | |  | 0.148 | | |  | 0.261 | | |  |  |  |  |  |  |  |  |  |
| RM Factor 1 ✻ group |  | 0.200 |  | 0.800 | |  | 0.131 | | |  | 0.869 | | |  | 1.656 | | |  |  |  |  |  |  |  |  |  |
|  | | | | | | | | | | | | | | | | | |  |  |  |  |  |  |  |  |  |
| **Total typed per min. (excl. spaces)** | | | | | | | | | | | | | | | | |  |  |  |  |  |  |  |  |  |  |
| **Effects** | **P(incl)** | | **P(excl)** | | | **P(incl\|data)** | | | | **P(excl\|data)** | | | | **BF_excl_** | | |  |  |  |  |  |  |  |  |  |  |
| RM Factor 1 |  | 0.600 |  | 0.400 | |  | 0.951 | | |  | 0.049 | | |  | 0.077 | | |  |  |  |  |  |  |  |  |  |
| group |  | 0.600 |  | 0.400 | |  | 0.990 | | |  | 0.010 | | |  | 0.016 | | |  |  |  |  |  |  |  |  |  |
| RM Factor 1 ✻ group |  | 0.200 |  | 0.800 | |  | 0.537 | | |  | 0.463 | | |  | 0.215 | | |  |  |  |  |  |  |  |  |  |
|  | | | | | | | | | | | | | | | | | |  |  |  |  |  |  |  |  |  |
| **Produced ratio (incl. spaces)** | | | | | | | | | | | | | | | | |  |  |  |  |  |  |  |  |  |  |
| **Effects** | **P(incl)** | | **P(excl)** | | | **P(incl\|data)** | | | | **P(excl\|data)** | | | | **BF_excl_** | | |  |  |  |  |  |  |  |  |  |  |
| RM Factor 1 |  | 0.600 |  | 0.400 | |  | 0.356 | | |  | 0.644 | | |  | 2.715 | | |  |  |  |  |  |  |  |  |  |
| group |  | 0.600 |  | 0.400 | |  | 0.454 | | |  | 0.546 | | |  | 1.803 | | |  |  |  |  |  |  |  |  |  |
| RM Factor 1 ✻ group |  | 0.200 |  | 0.800 | |  | 0.050 | | |  | 0.950 | | |  | 4.706 | | |  |  |  |  |  |  |  |  |  |
|  | | | | | | | | | | | | | | | | | |  |  |  |  |  |  |  |  |  |
| **Characters (incl. spaces)** | | | | | | | | | | | | | | | | |  |  |  |  |  |  |  |  |  |  |
| **Effects** | **P(incl)** | | **P(excl)** | | | **P(incl\|data)** | | | | **P(excl\|data)** | | | | **BF_excl_** | | |  |  |  |  |  |  |  |  |  |  |
| RM Factor 1 |  | 0.600 |  | 0.400 | |  | 0.355 | | |  | 0.645 | | |  | 2.720 | | |  |  |  |  |  |  |  |  |  |
| group |  | 0.600 |  | 0.400 | |  | 0.481 | | |  | 0.519 | | |  | 1.620 | | |  |  |  |  |  |  |  |  |  |
| RM Factor 1 ✻ group |  | 0.200 |  | 0.800 | |  | 0.049 | | |  | 0.951 | | |  | 4.845 | | |  |  |  |  |  |  |  |  |  |
|  | | | | | | | | | | | | | | | | | |  |  |  |  |  |  |  |  |  |
| **Characters (excl. spaces)** | | | | | | | | | | | | | | | | |  |  |  |  |  |  |  |  |  |  |
| **Effects** | **P(incl)** | | **P(excl)** | | | **P(incl\|data)** | | | | **P(excl\|data)** | | | | **BF_excl_** | | |  |  |  |  |  |  |  |  |  |  |
| RM Factor 1 |  | 0.600 |  | 0.400 | |  | 0.223 | | |  | 0.777 | | |  | 5.229 | | |  |  |  |  |  |  |  |  |  |
| group |  | 0.600 |  | 0.400 | |  | 0.457 | | |  | 0.543 | | |  | 1.781 | | |  |  |  |  |  |  |  |  |  |
| RM Factor 1 ✻ group |  | 0.200 |  | 0.800 | |  | 0.025 | | |  | 0.975 | | |  | 9.571 | | |  |  |  |  |  |  |  |  |  |
|  | | | | | | | | | | | | | | | | | |  |  |  |  |  |  |  |  |  |
| **Words** | | | | | | | | | | | | | | | | | | | | | | |  |  |  |  |
| **Effects** | **P(incl)** | | **P(excl)** | | | **P(incl\|data)** | | | | **P(excl\|data)** | | | | **BF_excl_** | | | | | | | | |  |  |  |  |
| RM Factor 1 |  | 0.600 |  | 0.400 | |  | 1.000 | | |  | 2.273×10^-4^ | | | |  | 3.410×10^-4^ | | | | | | | | |  | |
| group |  | 0.600 |  | 0.400 | |  | 0.678 | | |  | 0.322 | | | |  | 0.713 | | | | | | | | |  | |
| RM Factor 1 ✻ group |  | 0.200 |  | 0.800 | |  | 0.164 | | |  | 0.836 | | | |  | 1.278 | | | | | | | | |  | |
|  | | | | | | | | | | | | | | | | | | | | | | | | |  |  |
